# Long-Term Waterlogging as Factor Contributing to Hypoxia Stress Tolerance Enhancement in Cucumber: Comparative Transcriptome Analysis of Waterlogging Sensitive and Tolerant Accessions

**DOI:** 10.3390/genes12020189

**Published:** 2021-01-28

**Authors:** Kinga Kęska, Michał Wojciech Szcześniak, Izabela Makałowska, Małgorzata Czernicka

**Affiliations:** 1 Department of Plant Biology and Biotechnology, Faculty of Biotechnology and Horticulture, University of Agriculture in Krakow, Al. 29 Listopada 54, 31-425 Krakow, Poland; 2 Institute of Human Biology and Evolution, Faculty of Biology, Adam Mickiewicz University, Uniwersytetu Poznańskiego 6, 61-614 Poznań, Poland; miszcz@amu.edu.pl (M.W.S.); izabel@amu.edu.pl (I.M.)

**Keywords:** *Cucumis sativus* L., DEGs, gene expression, hypoxia, priming, RNA-Seq, transcriptome, waterlogging

## Abstract

Waterlogging (WL), excess water in the soil, is a phenomenon often occurring during plant cultivation causing low oxygen levels (hypoxia) in the soil. The aim of this study was to identify candidate genes involved in long-term waterlogging tolerance in cucumber using RNA sequencing. Here, we also determined how waterlogging pre-treatment (priming) influenced long-term memory in WL tolerant (WL-T) and WL sensitive (WL-S) i.e., DH2 and DH4 accessions, respectively. This work uncovered various differentially expressed genes (DEGs) activated in the long-term recovery in both accessions. De novo assembly generated 36,712 transcripts with an average length of 2236 bp. The results revealed that long-term waterlogging had divergent impacts on gene expression in WL-T DH2 and WL-S DH4 cucumber accessions: after 7 days of waterlogging, more DEGs in comparison to control conditions were identified in WL-S DH4 (8927) than in WL-T DH2 (5957). Additionally, 11,619 and 5007 DEGs were identified after a second waterlogging treatment in the WL-S and WL-T accessions, respectively. We identified genes associated with WL in cucumber that were especially related to enhanced glycolysis, adventitious roots development, and amino acid metabolism. qRT-PCR assay for hypoxia marker genes i.e., *alcohol dehydrogenase* (*adh*), *1-aminocyclopropane-1-carboxylate oxidase* (*aco*) and *long chain acyl-CoA synthetase 6* (*lacs6*) confirmed differences in response to waterlogging stress between sensitive and tolerant cucumbers and effectiveness of priming to enhance stress tolerance.

## 1. Introduction

Among the negative effects of climate change are more frequent violent downpours that cause local flooding, which, in turn, lead to long-term waterlogging of plants. As a consequence, this means a reduction in oxygen availability in the plant root zone, i.e., hypoxia [1,2,3]. Additional factors that trigger long-term hypoxia in the root system are compacted soil and poor substrate aeration [4], even in drought areas [5]. Hypoxic stress in plants may also occur in hydroponic greenhouse cultivation, where oxygen availability decreases due to inadequate irrigation of the root system and insufficient aeration of the flowing medium [6].

Cucumber (*Cucumis sativus* L.), due to its shallow root system [7], is considered as being sensitive to the stress of limited oxygen access [6,8,9,10]. Differences in the response to oxygen deprivation between waterlogging (WL) tolerant (WL-T) and WL sensitive (WL-S) accessions using the RNA-Seq approach were evaluated in *Arabidopsis thaliana* [11], rice (*Oryza sativa*) [12], rapeseed (*Brassica napus* L.) [13], soybean (*Glycine max*) [14], kiwifruit (*Actinidia deliciosa*) [15], sesame (*Sesamum indicum* L.) [16], and cucumber [17]. However, these studies focused mainly on the response to hypoxic stress in the initial stages; there are no data from RNA-Seq studies carried out in cucumber roots regarding long-term waterlogging in WL-S and WL-T accessions. Understanding the mechanisms involved in the response to hypoxia in tolerant genotypes will enable the development of cultivars resistant to excess water in the soil in areas at risk of waterlogging [10].

When the stress is over, the plant shifts into the recovery period [18]. Yeung et al. [19] proposed a signaling network that participates in the recovery period after waterlogging in plants. According to these authors, ROS (Reactive Oxygen Species), ABA (Abscisic acid) and ethylene are the first signals evoking various pathways during the recovery period. In turn, increased activity of SOD (Superoxide dismutase) and CAT (Catalase) was demonstrated in cucumber leaves in WL-T cucumber, whereas in WL-S activity of those enzymes was decreased after 7 days of waterlogging [20]. Nonetheless, the molecular mechanisms involved in the long-term recovery period after waterlogging in cucumber roots are still unknown. We wanted to better understand the pathways engaged in a long-term root recovery period in WL-S and WL-T cucumber accessions, which is very important in the context of future research on plant waterlogging tolerance [18,20].

It is known that plants have a stress memory that facilitates them overcoming recurring exposure to stress [21]. Primed plants, as a consequence of acquired stress memory [22], are more tolerant and able to respond faster and more effectively when they are exposed to stress factors again [23,24,25].

In cucumber, there are only a few papers describing the phenomenon of priming. Usage of melatonin as a priming factor has been studied against salt and water stress [26,27] and *Rhizobacterium* strains have been used against drought stress [28]. Still, the usage of waterlogging as a priming factor against oxygen deprivation in cucumber is not known. Priming and stress memory, as a consequence, are one of the main approaches developed by breeders for use in resistant plant production [29,30,31], either as an alternative to genetically modified (GM) plants or in beneficial financial terms [32]. Still, many questions remain unanswered, even those concerning the duration of memory in the life of a plant or the application of priming in the field conditions, not only in the laboratory.

In this work, we performed transcriptomic analysis using RNA-Seq to identify metabolic pathways regulated under long-term waterlogging treatment in both WL-T DH2 and WL-S DH4 cucumber accessions. Secondly, we wanted to determine how priming (pre-treatment) influences long-term memory in WL-T and WL-S cucumbers. Additionally, this work aimed to assess the effect of waterlogging on the long-term recovery period in cucumber roots.

## 2. Materials and Methods

### 2.1. Plant Material and Experiment Conditions

Seeds of two double-haploid (DH) lines of cucumber, i.e., DH2 (WL-T) and DH4 (WL-S), provided by KHiNO Polan (Poland), with contrasting responses to oxygen deprivation in the soil [10], were sown in 40-cell multi-pots (0.23 dm^3^ each cell) which were filled with Klasmann KTS-2 peat substrate (Klasmann; Geeste, Germany) containing (in mg dm^−3^) 250–500 N, 170–230 P_2_O_5_, 320–500 K_2_O, and 80–120 Mg. The plants were cultivated under controlled conditions in a greenhouse and were lit with supplementary radiation (high pressure sodium lamps) to sustain a 16/8 h light/dark regime at 27 °C during the day and 24 °C during the night. Minimum photosynthetic photon flux density (PPFD) on plant level during the day was 80 ± 20 µmol m^−2^ s^−1^. All plants were fertilized with growth fertilizer 3 days before the first waterlogging and every 3 days from the end of the first waterlogging stress with regenerative fertilizer. The growth fertilizer contained 7.5 g of Superba^TM^ Green Forte (8.2% N, 11.5% P_2_O_5_, 36.1% K_2_O, 2.8% MgO, 5.7% S, 0.23% Fe, 0.14% Mn, 0.03% Zn, 01% Cu, 0.04 B, 0.003% Mo) (Yara International ASA; Oslo, Norway), 7.0 g of YaraLiva CALCINIT Flakes (15.2% N, 27.5% CaO) (Yara International ASA; Oslo, Norway), and 3.0 g KRISTA^TM^ MAG (11% N, 15% MgO) (Yara International ASA; Oslo, Norway) diluted in 10 dm^3^ H_2_O, whereas the regenerative fertilizer consisted of 11.5 g, 8.8 g, and 4.4 g, respectively, of the same components as the growth fertilizer. The pH of the fertilizers was adjusted to 5.8 with nitric acid (V), and the final soil electrical conductivity (EC) was 2.8–3.1 ms cm^−1^; 25 mL of fertilizer was applied to each pot.

### 2.2. Stress Treatment

**Figure 1:**
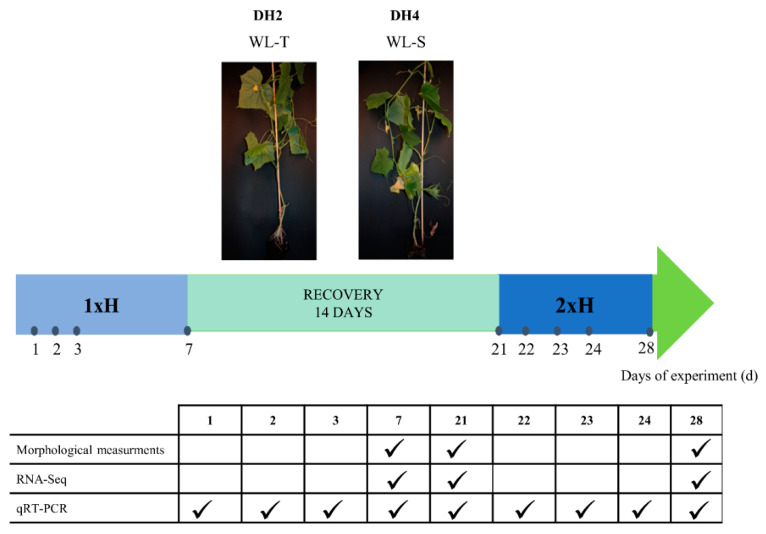
Scheme describing the experiment duration and time-points of sample collection for morphological parameter measurements, RNA-Seq and qRT-PCR. The image presents double haploid (DH) lines of 1xH treatment i.e., WL-T DH2 and WL-S DH4.

After 21 days of cultivation, plants were divided into three groups: untreated plants, cultivated under optimal conditions (Ctrl), non-primed plants, waterlogged for 7 days only once, (1xH), plants after 7 days of waterlogging and 14 days of recovery (Rec), and primed plants waterlogged for 7 days and after 14 days of recovery, then waterlogged again (2xH) (Figure 1).

The percent volumetric water content (VWC) was measured using a Delta-T Devices SM150 soil moisture sensor kit (Delta-T Devices Ltd.; Cambridge, UK) before stress treatment and, if needed, plants were watered to obtain a soil moisture level up to 30%. The root zone and hypocotyls at a height of around 4–7 cm of the plants in the 1xH group and 2xH group were waterlogged for 7 days in deep plastic trays (600 × 400 × 200 cm^3^). Then, plants were taken out of the water and stayed unstressed for 14 days (Rec). Later on, half of the stressed plants from the 1xH group were waterlogged for the second time for another 7 days (2xH) (Figure 1). The plants from the control group (Ctrl) stayed unstressed throughout the experiment and were watered as needed to ensure optimal growth conditions. Oxygen levels in the water (for waterlogged plants) and in the air (for control plants) were periodically monitored using a dissolved oxygen (DO) meter (HI 2040-02 edge, Hanna instruments; Woonsocket, RI, USA). During waterlogging, the oxygen level in the water reached 2.5 mg dm^−3^, which confirmed hypoxic condition [33], whereas in the air, the dissolved O_2_ level was 9.0 mg dm^−3^.

### 2.3. Measurement of Morphological Parameters

Effect of waterlogging stress on vegetative growth was estimated by comparing growth parameters between control and stressed plants. Before the waterlogging treatment, 20 random plants from three experimental groups i.e., Ctrl, 1xH, and 2xH, were labeled for plant height and leaf number. Plant height [cm], number of leaves, and root and shoot fresh weight (FW, g) and dry weight (DW, g) were measured at 7, 21, and 28 d of the experiment. Plant heights were measured from the base of the plant (above-ground) to the meristem. The weight of adventitious roots [g] (if developed) was also estimated. For the dry weight (DW) determination, the plant material was dried at 105 °C in an oven for 24 h and weighed afterwards.

### 2.4. RNA Extraction for RNA-Seq and qRT-PCR

For RNA-Seq analysis, the roots of both cucumber DH lines were collected from Ctrl, 1xH, Rec, and 2xH plants (Figure 1). Material from the control group was collected at 7, 21, and 28 d of the experiment and was pooled into 3 biological replicates, whereas single replicate contained the roots from 5 independent plants from each time point. For stressed plant groups (1xH, Rec, and 2xH), 3 biological replicates were prepared, whereas each of them pooled roots from 5 plants.

For the qRT-PCR assay, the roots were collected at 1, 2, 3, and 7 d from the control, 1xH, Rec, and 2xH groups at each time-point of the experiment.

Roots were collected, washed carefully in clean water, frozen immediately in liquid nitrogen, and then stored at −80 °C until RNA extraction. Total RNA isolation was performed with Direct-zol RNA MiniPrep Plus (Zymo Research, Irvine, CA, USA) according to the manufacturer’s instruction. All RNA extracts were treated with 1 U µL^−1^ RNase-free *Dnase* I (Thermo Fisher Scientific, Waltham, MA, USA) and 40 U µL^−1^ of RiboLock RNase Inhibitor (Thermo Fisher Scientific, Waltham, USA) to prevent DNA contamination. RNA quality and quantity were monitored by gel electrophoresis under denaturing conditions. The A260/A280 ratio and RNA integrity number (RIN) were determined by a Bioanalyzer 2100 (Agilent 2100 Bioanalyzer; Agilent Technologies, Palo Alto, Santa Clara, CA, USA).

### 2.5. RNA Library Construction and Illumina Sequencing

The cDNA libraries were prepared using the NEBNext^®^ Ultra^TM^ RNA Library Kit (Illumina, San Diego, CA, USA). In total, 24 cDNA libraries, i.e., two cucumber accessions (WL-T DH2 and WL-S DH4), x4 treatments (Ctrl, 1xH, Rec, 2xH), x3 experimental triplicates, were subjected to sequencing in PE101 (paired ends mode, with 101 bp read length) on an Illumina HiSeq4000 (Illumina, San Diego, CA, USA).

The RNA-Seq datasets generated for this study are deposited in the NCBI under BioProject PRJNA678740 and can be found in the GenBank Short Read Archive (SRA) under Acc. No. SSR13083584 to SSR13083607.

### 2.6. Data Filtering and Quality Control

The raw sequences in FASTQ format were subjected to TRueSeq3-PE adaptor removal using Cutadapt ver. 1.9.1 (http://cutadapt.readthedocs.io). Quality trimming was processed using BBDuk2 from the BBMap toolkit ver. 37.02 (https://jgi.doe.gov/data-and-tools/bbtools). FASTQC ver. 0.11.5, (https://www.bioinformatics.babraham.ac.uk/projects/fastqc/) was applied for standard pre- and post-trimming quality control. To pass the quality filter, read quality needed to surpass a Phred score (Q) of 20 and achieve a minimal length of 50 bp; all unpaired reads were excluded. *C. sativus* rRNA sequences were downloaded from the 5s rRNA database (http://combio.pl/rrna) and Bowtie2 ver. 2.3.3.1 (http://bowtie-bio.sourceforge.net/bowtie2/index.shtml) was employed for mapping and discarding 5s rRNA sequences from the set of data.

### 2.7. Mapping to the Reference Genome

The high-quality reads were aligned using STAR ver. 2.5.3a [34] (parameters: outSAMmapqUnique: 50; outSAMattributes: all; outSAMtype BAM SortedByCoordinate; outSAMstrandField: intronMotif; outFilterIntronMotifs: RemoveNoncanonical; outFilterType: BySJout; outFilterMultimapNmax: 20; alignSJoverhangMin: 8; alignSJDBoverhangMin: 1; alignIntronMin: 20; alignIntronMax: 1,000,000; alignMatesGapMax: 1,000,000; chimSegmentMin: 12; chimJunctionOverhangMin: 12; chimSegmentReadGapMax: 3) to the reference genome (ftp://cucurbitgenomics.org/pub/cucurbit/genome/cucumber/Chinese_long/v2) downloaded from the Cucurbit Genomics Database at http://cucurbitgenomics.org published by Huang et al. [35]. The alignment data were used to calculate the distribution of reads for the reference genes and to perform coverage analysis.

### 2.8. Transcriptome de novo Assembly

De novo transcriptome assembly for each biological replicate was performed using StringTie ver.1.3.3b [36] with the following parameters: minimum assembled transcript length: 200, minimum reads per bp coverage: 10, minimum junction coverage: 10. Cuffmerge and Cuffdiff programs, parts of the Cufflinks package ver.2.2.1 (http://cole-trapnell-lab.github.io/cufflinks) were used to merged the reference annotation and to estimate the transcript abundance. TransDecoder program ver. 5.0.1 [37] was used to identify the coding regions within the transcripts.

### 2.9. Differential Expression Estimation

**Figure 2:**
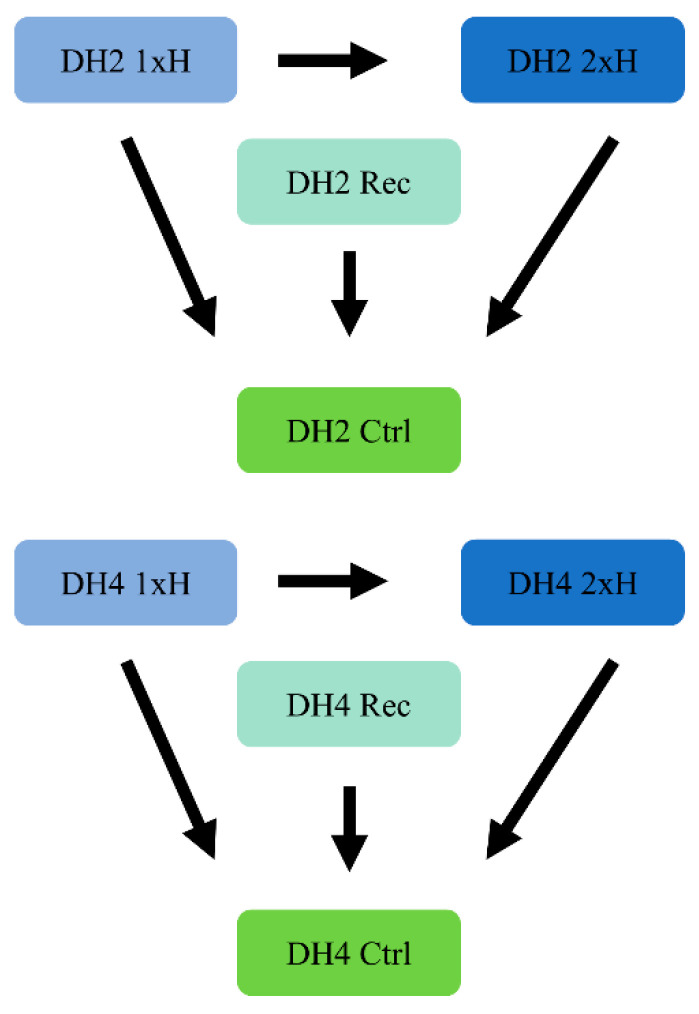
Strategy for differentially expressed genes (DEGs) identification. The arrow direction depicts the comparisons carried out in the analysis.

Gene expression levels for each sample were estimated by RSEM ver. 1.3.0 (https://deweylab.github.io/RSEM) [38]. Differentially expressed genes were identified using two R packages, i.e., edgeR ver. 3.20.1 [39] and DeSeq2 ver. 1.18 [40] using the p-value correction by Benjamini and Hochberg for the number of repetitions. For further analysis, transcripts identified both as differentially expressed in edgeR and DeSeq2, considering FDR (false discovery rate) < 0.05, were selected (Supplementary Materials Figure S1). Differential gene expression analysis was performed according to the scheme presented in Figure 2. One of the main goals was to compare responses to waterlogging stress between WL-S DH4 and WL-T DH2 treated once (1xH), for that purpose, we compared DH2 1xH vs. DH2 Ctrl and DH4 1xH vs. DH4 Ctrl. Secondly, we wanted to identify DEGs regulated by the second waterlogging treatment (2xH), so that we performed following comparisons: DH2 2xH vs. DH2 Ctrl, DH4 2xH vs. DH4 Ctrl, DH2 1xH vs. DH2 2xH, and DH4 1xH vs. DH4 2xH. Additionally, we wanted to determine changes at the transcriptomic level in plants after 14 days of recovery, so we compared DH2 Rec vs. DH2 Ctrl and DH4 Rec vs. DH4 Ctrl.

### 2.10. Functional Analysis

The putative function of assembled transcripts was determined using the Trinotate annotation pipeline ver. 3.1.0 (https://trinotate.github.io), dedicated to de novo assembled transcriptomes [41]. The protein-coding regions in assembled unigenes were predicted using TransDecoder ver. 5.0.1 [37]. The pipeline included searching for nucleotide (BLASTx) and protein (BLASTp) homology, performed against the UniProtKB/Swiss-Prot database [42]. We performed the identification of functional protein domains (HMMER/PFAM) [43,44], the prediction of potential protein signals and transmembrane domains (SignalP/tmHMM) [45]) and a comparison with gene annotation databases, i.e., the eggNOG [46], Gene Ontology (GO) [47], and Kyoto Encyclopedia of Genes and Genomes (KEGG) databases (http://www.genome.jp/kegg/) [48]. All annotations were loaded into the Trinotate SQLite database to generate the final annotation report. The topGO R/Bioconductor package ver. 2.38.1 [49] was implemented to test enrichment annotation terms. The significance of occurrence for a certain GO term was determined using Fisher’s exact test with a classic method. Analysis focused on groups of genes enriched for the biological process (BP), molecular function (MF), and cellular compartment (CC) gene ontologies, considering gene groups as significantly regulated with *p*-values ≤ 0.05. Genes associated with the response to oxygen deprivation in DH2 and DH4 cucumbers were investigated for gene ontological enrichment of biological processes with a *p*-value < 0.05 as the cut-off criterion using the R package clusterProfiler ver. 3.6.0 [50] (http://www.bioconductor.org/packages/release/bioc/html/clusterProfiler.html).

### 2.11. qRT-PCR Assay

For the qRT-PCR assay, we selected genes regulated by a limited supply of oxygen and in addition associated with hypoxia tolerance in plants [51,52,53]. Among selected genes were *alcohol dehydrogenase* (*adh*), a gene involved in fermentation, *1-aminocyclopropane-1-carboxylate oxidase* (*aco*), associated with the synthesis of ethylene and involved in adventitious root development, and *long chain acyl-CoA synthetase 6* (*lacs6*), involved in fatty acid metabolism. The purpose of qRT-PCR was to monitor the expression level of these genes throughout the treatment to firstly confirm the hypoxic conditions, and second to evaluate differences between cucumber accessions and depict the highest expression level over time. qRT-PCR was performed on 1, 2, 3, and 7 d plants treated once (1xH) and twice (2xH), and in plants in the Rec group, at 21, 22, 23, 24, and 28 d of the experiment. cDNA was obtained from 1 μg of total RNA using the iScript^TM^ cDNA Synthesis Kit (Bio-Rad laboratories, Hemel Hempstead, UK) following the manufacturers’ instructions. cDNA was diluted 1:5 with DNase/RNase-free H_2_O and stored at −20 °C. Quantitative real-time PCR was performed with a QuantStudio 3 Real-Time PCR System (Applied Biosystems, Foster City, CA, USA). A single technical repeat contained (25 µL) 12.5 μL of Maxima™ SYBR Green/ROX qPCR Master Mix (2X) (Thermo Scientific, Waltham, CA, USA), 5 µM of gene-specific forward and reverse primers, 1.5 μL of cDNA and RNase-free H_2_O. All qPCR reactions were run as three biological replicates, each with three technical replicates. No-template controls (NTCs) were included in every qPCR run. The qPCR method described above conforms to the MIQE (The Minimum Information for Publication of Quantitative Real-Time PCR Experiments) guidelines [54]. The qPCR reactions were amplified at 95 °C for 10 min, followed by 40 cycles of 95 °C for 15 s and 60 °C for 1 min., with a final dissociation curve analysis to check for the specificity of amplification. Relative quantification of gene expression was calculated according to the qBase method [55]. *Actin* (*act*) and *tubulin α chain* (*tua*) were used as the endogenous reference genes. The highly specific primers for *aco* and *lacs6* were designed using the IDT-PrimerQuest tool (http://eu.idtdna.com/primerquest/home/index) and validated using IDT-OligoAnalyzer 3.1 (https://eu.idtdna.com/calc/analyzer) considering the absence of primer-dimers and hairpin structures. All the primers used in this study are listed in Supplementary Materials Table S1.

### 2.12. Statistical Analysis

Student’s *t*-test with *p* < 0.05 was used to compare changes in morphological parameters. Comparisons between treatment and control conditions were conducted at 7, 21, and 28 d of the experiment. Two-way analysis of variance (ANOVA) with two factors (time and treatment) followed by Tukey’s test (*p* ≤ 0.05) were conducted to determine the influence of hypoxia on gene expression levels. Data are presented as the mean value ± SD for each treatment and time point. The analysis was performed using Statictica ver. 12 (Statsoft).

## 3. Results

### 3.1. Plant Morphological Traits

**Figure 3:**
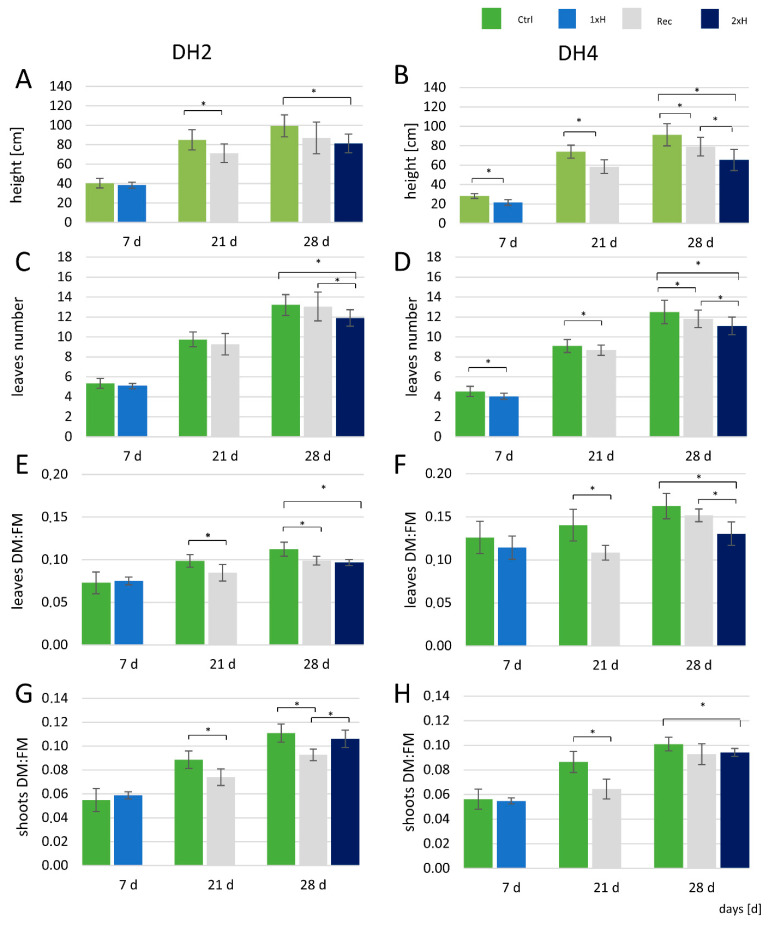
Effect of waterlogging on the plant height (**A**,**B**), leaves number (**C**,**D**), DM:FM ratio in leaves (**E**,**F**) and shoots (without leaves) (**G**,**H**) in DH2 and DH4 cucumber accessions, respectively. DM—dry mass, FM—fresh mass. Values are mean ± standard deviation (*n* = 10 for plant height and leaf number, *n* = 6 for FM and DM). * Indicates a significant difference between the indicated treatments at *p* ≤ 0.05 according to Student’s *t*-test.

The DH2 and DH4 cucumber accessions were assessed as tolerant (WL-T) and sensitive (WL-S), respectively, after 7 days of hypoxia treatment [10]. Here, the aim of analysis of morphological traits was confirmation of differential stress response of both DH cucumber lines. The effects of waterlogging on plant morphology were monitored at 7, 21, and 28 d of the experiment. After 7 days of stress, a reduction in plant height and the number of leaves was found only in the DH4 accession (Figure 3A,B). In plants of the Recovery group (Rec), a decrease in plant height was observed compared to the non-waterlogged plants (Ctrl) in both cucumber lines (Figure 3A,B). A second treatment with waterlogging stress (2xH) resulted in slower plant growth in both cucumber accessions. At 28 days (after 3 weeks of recovery in 1xH plants), no difference in height was observed in WL-T (DH2). A negative effect of single (1xH) and double (2xH) waterlogging treatments on the number of leaves was observed in WL-S DH4, while a decrease in the number of leaves was recorded in WL-T DH2 only after treating the plants for second time with the stress (2xH) (Figure 3C,D). The number of leaves was lower in the Recovery group of WL-S DH4 compared to the non-stressed plants (Ctrl).

Results shown in Figure 3E–H demonstrate that there was no effect of a single waterlogging (1xH) treatment on the DM:FM ratio in leaves and shoots in the two cucumber accessions. There was a significant decrease in the DM:FM ratio in recovered plants (at 21 days of the experiment) in the DH2 and DH4 accessions. However, a second 7-day waterlogging treatment (2xH) in the two DH lines reduced the DM:FM ratio.

**Figure 4:**
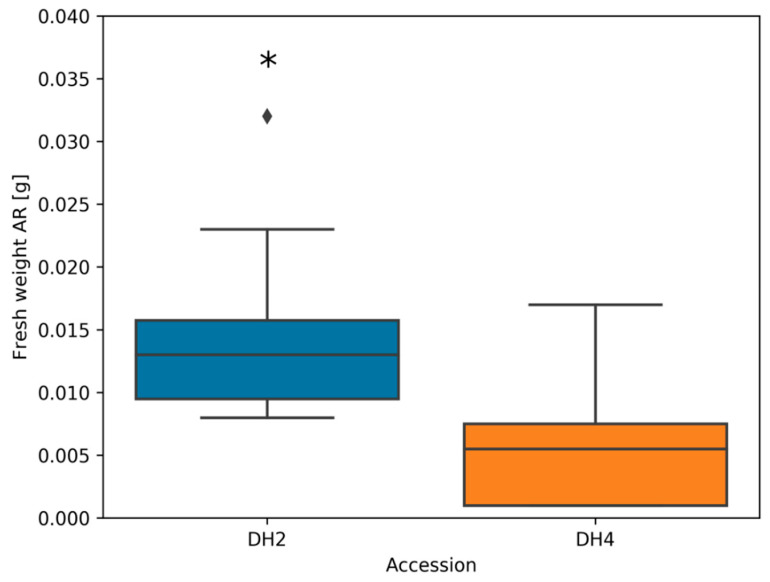
Mass of adventitious roots determined after 7 days of waterlogging in DH2 and DH4 cucumber accessions. Asterix indicates significant differences between accessions (*n* = 10) according to Student’s *t*-test (*p* < 0.05). The diamond represents the outlier.

In both DH lines, 7 days of waterlogging caused the development of adventitious roots. However, a greater mass of adventitious roots was found in WL-T DH2 compared to WL-S DH4 (Figure 4).

### 3.2. RNA Sequencing and Transcriptome Assembly

The pool of RNA-Seq reads included in total 1,156,427,650 reads (115.2 Gbp), of which 584,234,614 reads (50.5%) were represented by WL-T DH2 and 572,193,036 (49.5%) by WL-S DH4 (Supplementary Materials Table S2). In total, 1,026,182,014 (88.7%) were considered as clean reads.

### 3.3. Transcriptome Characterization and Functional Annotation

**Figure 5:**
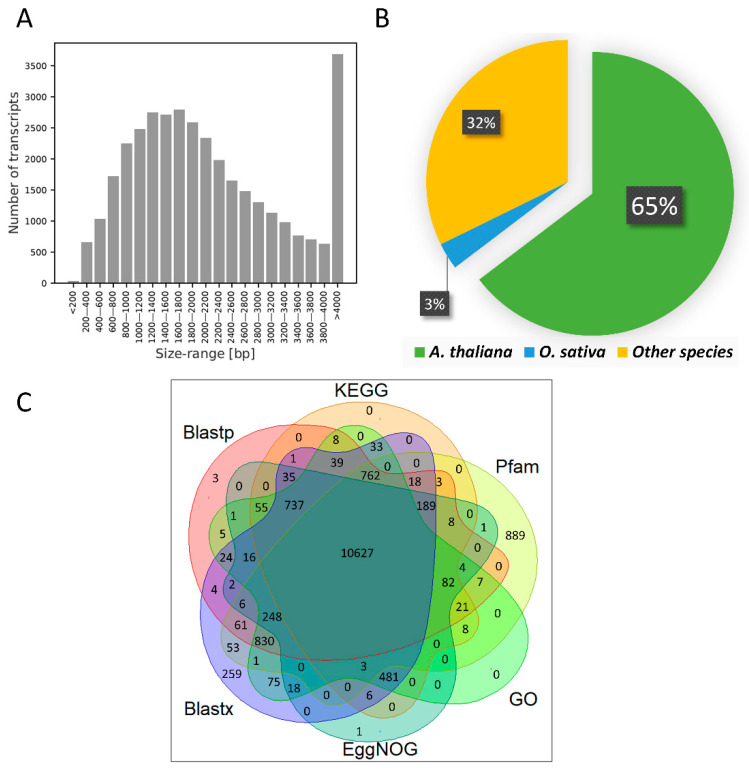
(**A**) Transcript size distribution. (**B**) Homologous species distribution. (**C**) Venn diagram displaying differences of annotation based on the Blastp, Blastx, KEGG, GO, EggNOG, and Pfam databases.

The clean reads represented all samples and were subjected to de novo assembly, resulting in 35,712 transcripts corresponding to 18,093 unigenes (Table 1). The length of the transcripts varied from 162 to 20,773 bp with an average length of 2236 bp. The transcript size distribution showed a high proportion of transcripts in the size range of 800–2200 bp (Figure 5A).

To identify the putative function of the assembled transcripts of *Cucumis sativus* L., sequence homology searches against the UniprotKB/SwissProt database using BLASTx and BLASTp displayed that 29,077 (81.42%) nucleotide sequences and 26,883 (75.28%) protein sequences were aligned (Supplementary Materials Table S3). Functional annotation of transcripts revealed that most of them (76%) have homologs in *Arabidopsis thaliana* (Figure 5B). Searching against the Pfam database resulted in the identification of 65,002 probable domains in the *C. sativus* transcriptome, of which 4047 were classified into unique domain groups. The most abundant domains were connected with the pentatricopeptide repeat (PPR) motif (PF01535.19, PF13041.5, PF13812.5, and PF12854.6). Only in 2285 and 6924 signal peptide transcripts and transmembrane regions were predicted with SignalP and TmHMM, respectively. A total of 15,623 were annotated in at least one database (Figure 5C).

**Figure 6:**
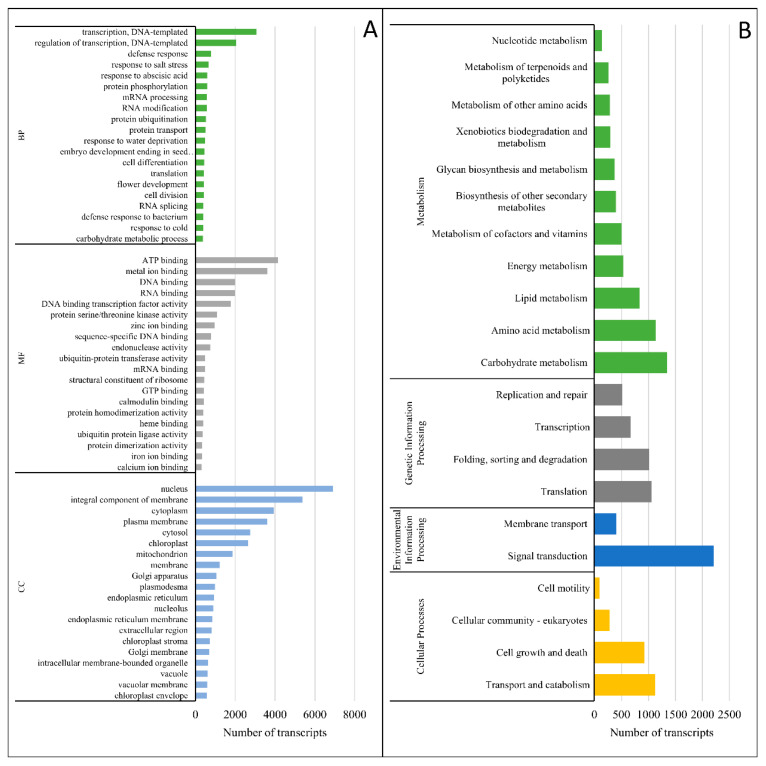
(**A**) Distribution of *C. sativus* transcripts in the top 20 gene ontology (GO) categories in biological processes (BP), molecular functions (MF), and cellular components (CC). (**B**) Pathway assignment was based on the Kyoto Encyclopedia of Genes and Genomes (KEGG) database. Classification was based on organismal system categories, cellular process categories, environmental information processing categories, genetic information processing categories, and metabolism categories.

Gene ontology (GO) functional analysis was performed to classify the assembled transcripts into three functional categories, i.e., biological processes (BP), molecular functions (MF), and cellular components (CC). In total, 27,573 (77.11%) out of all annotated transcripts were predicted with 7699, 4292, and 2517 gene ontology terms in BP, MF, and CC, respectively (Figure 6). The highly represented terms in all GO categories were presented in Figure 6A.

A total of 391 KEGG pathways were identified for 25,590 (71.7%) transcripts (Supplementary Materials Data S1). The annotated pathways were categorized into six main groups i.e., ‘organism systems’, ‘cellular processes’, ‘environmental information processing’, ‘genetic information processing’, and ‘metabolism’ (Figure 6B). The highest number of transcripts (387) was assigned to ‘plant hormone signal transduction pathway’ [ko04075] in the ‘environmental information processing’ category. In the ‘metabolism’ category, the most dominant pathways were ‘glycerophospholipid metabolism’ [ko00564] (189 transcripts), ‘amino sugar and nucleotide sugar metabolism’ [ko00520] (184 transcripts), and ‘purine metabolism’ [ko00230] (175 transcripts), involved in ‘lipid, carbohydrate, and nucleotide metabolism’, respectively.

### 3.4. Identification of Differentially Expressed Genes (DEGs)

In WL-T DH2, it was found that, under a single waterlogging treatment (1xH), 5957 genes were differently expressed, compared with untreated plants (Ctrl), including approximately 57% of the genes (3373) characterized by down-regulated expression and 43% (2584) of the genes showing over-expression (Table 2). After a 14-day recovery period (Rec), the number of DEGs decreased more than nine-fold, i.e., 654 DEGs were identified, of which 355 were overexpressed and 299 were diminished in expression compared to Ctrl. In plants treated a second time by the stress (2xH), 5007 significant DEGs were detected compared with the unstressed plants (Ctrl), of which 46% (2310) were positively expressed.

In the case of the WL-S DH4 accession, as a result of the first waterlogging stress (1xH), the number of genes with differential expression was 33% higher compared with WL-T DH2 i.e., 8927 DEGs were identified (Table 2). After the regeneration period (Rec) of waterlogged WL-S DH4 plants, the number of DEGs was reduced by nearly five-fold compared to 1xH plants (1877), of which 892 DEGs were up-regulated and 985 were down-regulated. The second stress (2xH) resulted in a further increase in the number of DEGs compared with unstressed plants (Ctrl), i.e., 11,619 DEGs were identified in total, which was the largest number of identified DEGs among the combinations. A list of all identified DEGs for all comparisons are presented in Supplementary Materials Data S2–S9.

### 3.5. Differential Response to Waterlogging of Primed and Non-Primed WL-T and WL-S Cucumbers

In this study, we wanted to examine if pre-treatment (priming) with waterlogging stress in two cucumber accessions with different stress tolerance would have an impact on the transcriptomic response to repeated treatment with waterlogging stress.

**Figure 7:**
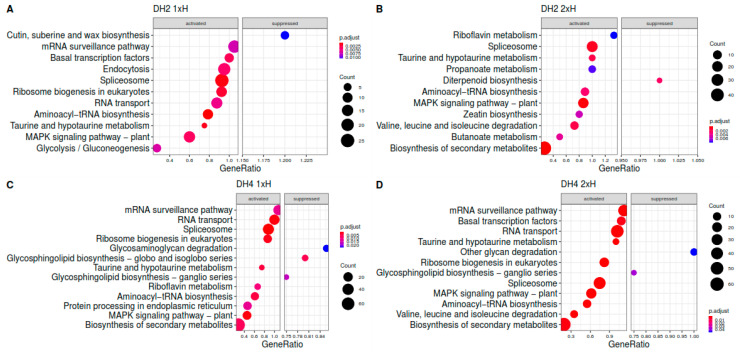
Scatter plot for the KEGG enrichment results obtained for WL-T DH2 (**A**,**B**) and WL-S (**C**,**D**) plants treated once (1xH) and twice (2xH) with waterlogging in comparison to untreated plants. The number of core genes (“count”) divided by the total number of genes is the gene ratio. The sizes of the dots represent the number of core genes, and the color indicates the adjusted *p*-value. Only pathways with *p*-values < 0.05 were eligible for enriched biological processes.

To identify the biological pathways activated in plants treated once (1xH) and twice (2xH) with hypoxic stress in WL-T DH2 and WL-S DH4 cucumbers, DEGs were mapped to the reference canonical pathways in KEGG (Figure 7). The common and specific pathways activated in both stress treatment i.e., 1xH and 2xH were indicated. In WL-T DH2 among specific biological pathways regulated in plants treated once with hypoxia (1xH), were activated i.e., ‘basal transcription factors’, ‘RNA transport’, ‘endocytosis’, ‘spliceosome’, and ‘ribosome biogenesis in eucaryotes’ assigned as activated pathways, whereas ‘cutin, suberine, and wax biosynthesis’ was suppressed (Figure 7A). The unique pathways in plants exposed to hypoxia stress for the second time (2xH) were also specified (Figure 7B). In this case, DEGs were assigned to the activation of ‘riboflavin metabolism’, ‘propanoate metabolism’, ‘zeatin biosynthesis’, ‘valine, leucine and isoleucine degradation’, ‘biosynthesis of secondary metabolites’, and suppression of ‘diterpenoid biosynthesis’.

In case of the WL-S DH4 accession, the specific pathways activated under hypoxia in plants after 7 days of waterlogging treatment (1xH) were: ‘circadian rhythm—plant’, ‘basal transcription factors’, ‘riboflavin metabolism’, whereas ‘glycosphingolipid biosynthesis—globo and isoglobo series’ was suppressed (Figure 7C). In plants waterlogged again after 14 days of recovery (2xH), we found specific activated pathways connected with ‘mRNA surveillance pathway’, ‘autophagy—other’ and ‘valine leucine and isoleucine degradation’ (Figure 7D). KEGG pathway analysis revealed that WL-S DH4 strongly activated the biosynthesis of secondary metabolites in response to 7 days of waterlogging (1xH), whereas this was not detected in the WL-T DH2 accession. Biosynthesis of secondary metabolites was activated only after the second treatment (2xH) in the WL tolerant cucumber (DH2).

In the 1xH WL-T DH2 plant group, the classification of DEGs into the specific metabolic pathways using the MapMan tool resulted in the 2998 DEGs mapped, of which 521 were visible in the metabolism overview scheme (Supplementary Materials Figure S2A,B). In contrast, in the 2xH plant group, 1629 DEGs were mapped and 297 DEGs were visible. In the DH4 cucumber, 2654 DEGs identified for 1xH were mapped, of which 489 DEGs were visible on the scheme, whereas in the 2xH plant group, 6341 DEGs were mapped, of which 1109 DEGs were assigned (Supplementary Materials Figure S2C,D).

DEGs identified in the 1xH and 2xH groups of both cucumber lines, involved in hormone metabolism, were mapped to the biosynthesis pathways associated with IAA, ABA, ethylene, cytokinins, and jasmonates. In WL-T DH2, the number of mapped genes was higher in plants treated once (1xH) in comparison to plants treated twice (2xH) (Supplementary Materials Figure S3A,B), whereas in WL-S DH4, the tendency was reverse, as a higher number of mapped genes involved in hormone metabolism were noted in plants waterlogged twice (2xH) (Supplementary Materials Figure S3C,D). The highest number of DEGs potentially involved in tolerance was assigned to the regulation of ABA and jasmonates.

**Figure 8:**
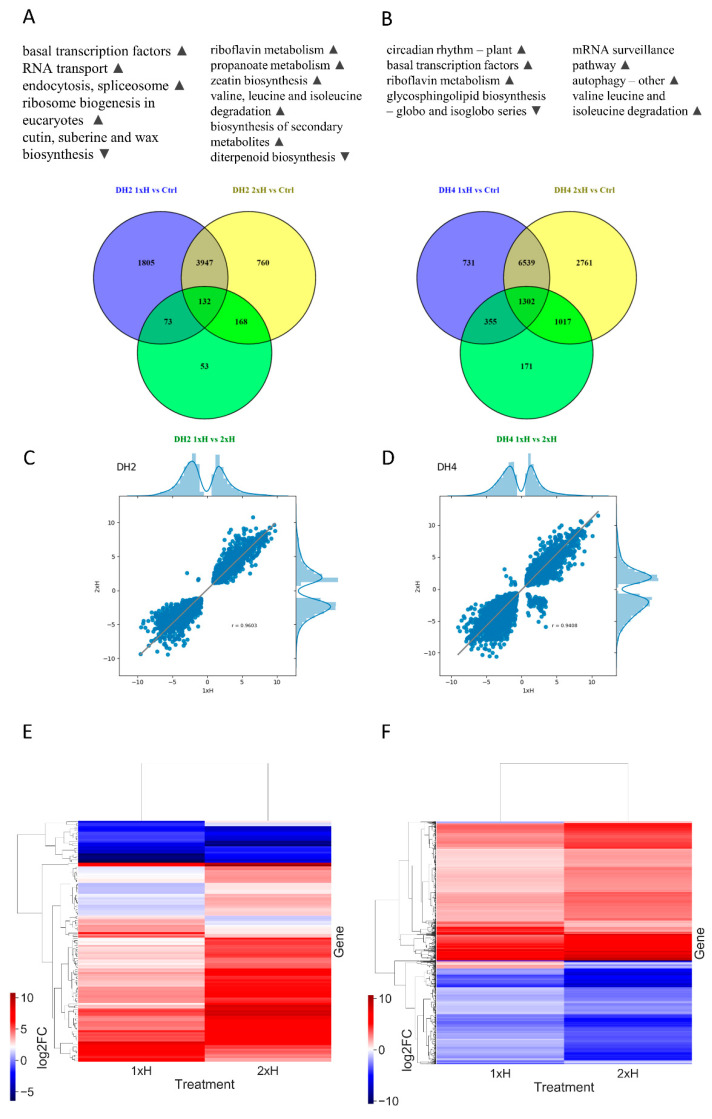
Number of differentially expressed genes (DEGs) specifically and commonly expressed in plants exposed to stress once (1xH) and twice (2xH) in the WL-T DH2 (**A**) and WL-S DH4 (**B**) accessions. Above the Venn diagrams, specific pathways for 1xH and 2xH treatment are listed. ▲ indicates up-regulation of pathway, ▼ indicates down-regulation of pathway. The correlation coefficient between 4079 and 7841 DEGs identified in plants treated once (1xH) and twice (2xH) in WL-T DH2 (**C**) and WL-S DH4 (**D**) is shown. Axes consist of Fold Change representing as ratio Treatment/Control. The heat map shows statistically significant DEGs commonly expressed in 1xH and 2xH in WL-T DH2 (**E**) and WL-S DH4 (**F**).

Among all DEGs, we identified commonly regulated DEGs with 1xH and 2xH treatment and simultaneous statistically differential expression level between 1xH and 2xH. For this purpose, we compared DEGs in plants waterlogged once (1xH) and plants exposed twice to that stress (2xH) in comparison to control conditions (Ctrl) and also between them (Figure 8A,B). We identified 4079 and 7841 DEGs commonly expressed in 1xH and 2xH in WL-S DH2 and WL-T DH4, respectively. Additionally, we found that the common DEGs had strongly correlated expression patterns in 1xH and 2xH because the correlation coefficients was 0.96 and 0.94 for DH2 and DH4, respectively (Figure 8C,D). However, among the DEGs commonly expressed in 1xH and 2xH, we selected 132 and 1302 differentially expressed genes between 1xH and 2xH in WL-T DH2 and WL-S DH4, respectively (Figure 8E,F). We found 3 and 34 DEGs with opposite expression patterns in 1xH and 2xH in WL-T DH2 and WL-S DH4, respectively. In WL-T DH2, these DEGs were annotated as *α-amylase/subtilisin inhibitor* (XLOC_003864), *β-amyrin 11-oxidase* (XLOC_006633), *probable isoaspartyl peptidase/L-asparaginase 2* (XLOC_00945), revealing down-regulation in 1xH (log_2_FC = −2.96, log_2_FC = −1.37, log_2_FC = −1.30, respectively) and up-regulation in 2xH (log_2_FC = 2.58, log_2_FC = 1.54, log_2_FC = 1.68, respectively) (Supplementary Materials Table S4).

In the case of the WL sensitive accession (DH4), five DEGs were down-regulated with a single waterlogging treatment (1xH), but up-regulated after second waterlogging treatment (2xH); these included *sugar transporter ERD6-like 16* (XLOC_014798), *calmodulin-like protein 8* (XLOC_002728), and *protein SMAX1-LIKE 4* (XLOC_000765). These DEGs are involved in biological processes such as ‘hexose transmembrane transport’ (GO:0035428), ‘detection of calcium ion’ (GO:0005513), and ‘carbohydrate homeostasis’ (GO:0033500) and may be involved in acquiring tolerance to waterlogging in cucumber. The reverse expression pattern was noted in 29 DEGs, where among others, up-regulation of DEGs after 1xH and down-regulation after a second treatment (2xH) were noted for *cinnamoyl-CoA reductase 1* (XLOC_014021), *calcium-binding protein PBP1* (XLOC_014985), *MLP-like protein 328* (XLOC_011942), and *probable glycosyl transferase* (XLOC_003800). These DEGs were assigned to the following biological processes: ‘lignin biosynthetic process’ (GO:0009809), ‘response to auxin’ (GO:0009733), ‘response to cytokinin’ (GO:0009735), and ‘cell wall organization’ (GO:0071555), respectively. Moreover, 14 of 34 DEGs were annotated as unknown (Supplementary Materials Table S4), but they were strongly regulated by waterlogging and may play important roles in the response to oxygen deprivation. These genes with opposite expression levels allowed us to distinguish cucumber plants treated once (1xH) from plants treated twice with waterlogging (2xH).

**Figure 9:**
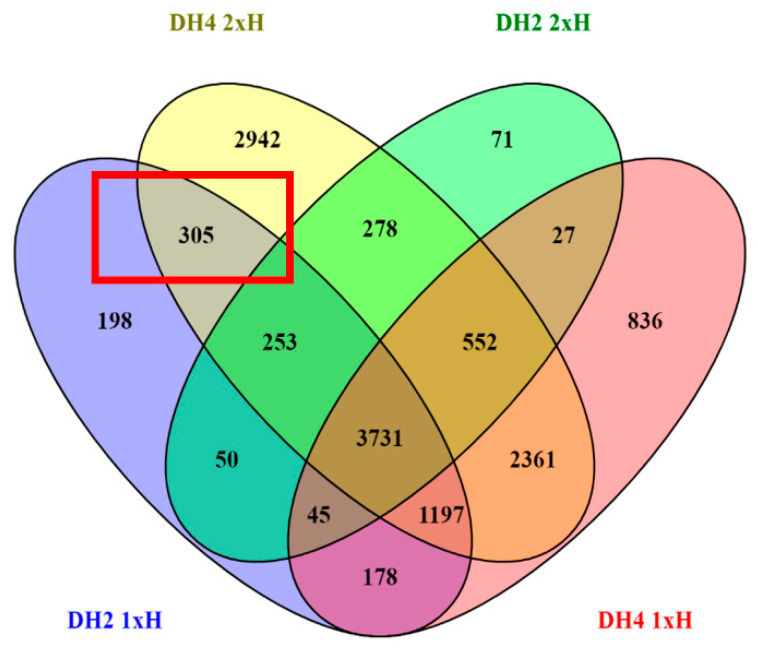
Venn diagram displaying common and specific DEGs regulated under 1xH and 2xH treatment in comparison to control conditions.

Since accession DH2 is considered to be WL tolerant, we wanted to find DEGs uniquely regulated in WL-T after 1xH and in WL-S after the second exposure to stress (2xH) in order to indicate DEGs involved in enhanced tolerance to oxygen deprivation. As result, we identified 305 DEGs exclusively regulated both in the WL-T DH2 cucumber once waterlogged (1xH) in comparison to control plants and as well as in WL-S DH4 plants after the second stress treatment (2xH) (Figure 9). We found, among other DEGs, that *GDSL esterase/lipase*, *aspartate aminotransferase*, *mitochondrial*, *glutamate decarboxylase 3*, *triosephosphate isomerase*, *cytosolic*, *sucrose synthase*, and *expansin-like A1* were strongly up-regulated under hypoxic conditions in tolerant plants (WL-T) after a single waterlogging (1xH) and in the WL sensitive accession after the second treatment (2xH), meaning that the WL-S accession after the second treatment induced the expression of genes potentially connected with tolerance to oxygen deprivation (Supplementary Materials Data S10).

### 3.6. DEGs Determined in WL-T DH2 and WL-S DH4 Cucumbers in Recovery

To determine pathways and genes regulated in long-term recovery period after hypoxic stress in the WL-T DH2 and WL-S DH4 accessions, we analyzed the comparison Rec vs. Ctrl in both DH lines. Gene ontology (GO) enrichment of the DEGs identified in WL-T DH2 and WL-S DH4 were presented as Supplementary Materials Tables S5 and S6, respectively.

**Figure 10:**
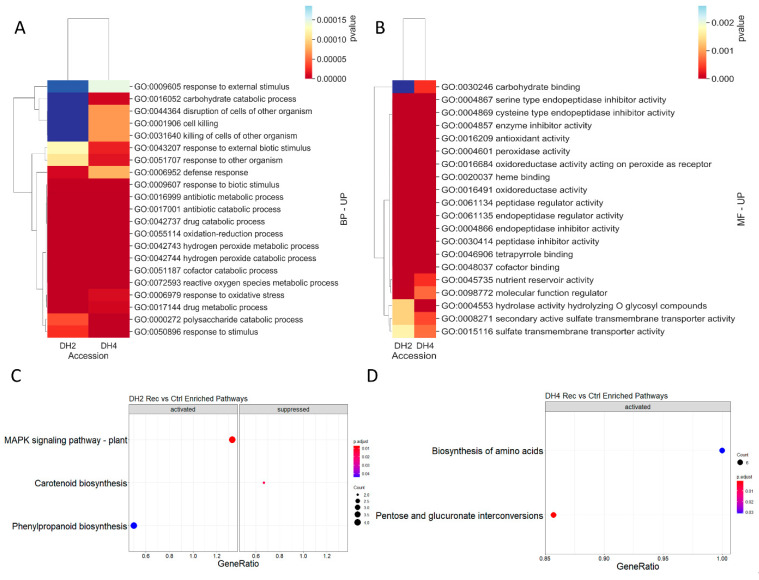
GO terms commonly enriched into (**A**) biological processes and (**B**) molecular functions after the recovery period in the DH2 and DH4 lines. Scatter plot for KEGG enrichment results obtained for plants after the recovery period in comparison to untreated plants in the DH2 (**C**) and DH4 (**D**) lines. The number of core genes (“count”) divided by the total number of genes is the gene ratio. The sizes of the dots represent the number of core genes, and the color indicates the adjusted *p*-value. Only pathways with *p*-values < 0.05 were eligible for enriched biological processes.

Considering common BP and MF terms in WL-T (DH2) and WL-S (DH4), 21 terms in the BP category and 20 terms in the MF category were indicated, respectively (Figure 10A,B). For both the BP and MF terms, the most differentiating ones were processes connected with carbohydrate metabolism, i.e., carbohydrate catabolic process (GO:0016052) and carbohydrate binding (GO:0030246), which were significantly altered in WL-S DH4 (Figure 10B). Among the pool of unique DEGs involved in carbohydrate metabolism in WL-S DH4 were *probable pectate lyase 22*, *β-amylase 3*, *multiple inositol polyphosphate phosphatase 1*, *putative glucose-6-phosphate 1-epimerase*, *β-galactosidase 3*, *endoglucanase 6*, *naringenin*, *2-oxoglutarate 3-dioxygenase*, *ACC oxidase 1*, and *ACC oxidase 3* and two DEGs in WL-T DH2, i.e., *pyruvate dehydrogenase E1 component subunit α* and *F-box protein PP2-B15*.

For DEGs identified in the WL-T DH2 accession, KEGG analysis indicated pathways related to ‘MAPK signaling pathway—plant’, ‘phenylpropanoid biosynthesis’ and ‘carotenoid biosynthesis’ (Figure 10C). In the WL-S DH4 accession, two significantly activated pathways were identified, i.e., ‘pentose and glucoronate interconversion’ and ‘biosynthesis of amino acids’ (Figure 10D).

### 3.7. qRT-PCR Analysis

**Figure 11:**
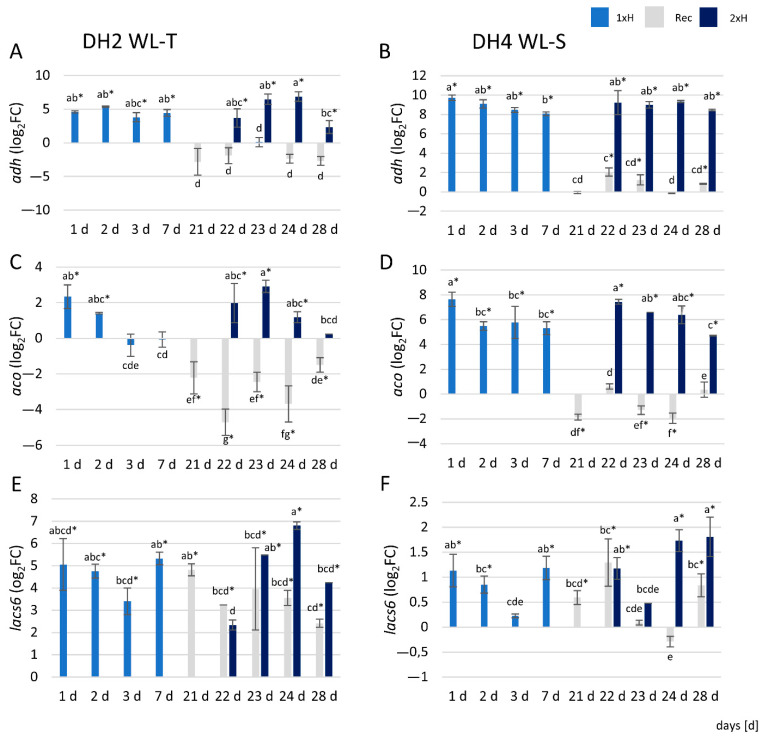
Expression profiles of the *adh* (**A**,**B**), *aco* (**C**,**D**), and *lacs6* (**E**,**F**) genes in WL-T DH2 and WL-S DH4 cucumber accessions under waterlogging stress, respectively. Data are expressed as the mean ± SD (standard deviation) of three independent biological replicates and three technical replications with *p* < 0.05 (Tukey’s post-hoc test). Asterisks indicate a significant difference vs. control plants. The same letters indicate no statistical differences between treatments and time-points (days).

The results show that the expression level of genes, i.e., *alcohol dehydrogenase* (*adh*), 1-aminocyclopropane-1-carboxylate oxidase (*aco*), and *long-chain acyl-CoA synthases 6* (*lacs6*) involved in response to hypoxia were differentially regulated between the WL-T and WL-S cucumber DH lines. The relative expression level of the *adh* gene was approximately two-fold higher in WL-S DH4 plants under waterlogging stress than in WL-T DH2 plants (Figure 11A,B). In WL-T DH2, after the second waterlogging (2xH), *adh* gene expression increased from day 1 to 3 (22–24 days of the experiment), then decreased, while in WL-S DH4, the *adh* gene was up-regulated to the same level throughout the 7 days of stress.

The expression level of the *aco* gene was significantly increased after 1 d of waterlogging treatment in the 1xH group of both cucumber lines; however, in WL-S DH4, the expression level was 2.5 times higher than in WL-T DH2. Up-regulation of the *aco* gene in WL-T DH2 was detected at 1 and 2 d after the first (1xH) and second (2xH) waterlogging treatments. Subsequently, expression declined, and at 7 d was comparable to control. In contrast, in WL-S DH4, up-regulation of the *aco* gene was maintained at the same level during the 7 days of the first (1xH) and second (2xH) waterlogging treatments. In recovered plants (Rec), the *aco* gene was down-regulated in both lines (Figure 11C,D).

Waterlogging treatment enhanced the expression of the *lacs6* gene just 1 d after stress induction in both cucumber lines; however, in WL-T DH2, up-regulation of the *lacs6* gene was meaningful, compared to the expression level detected in WT-S DH4. After a second waterlogging treatment (2xH), *lacs6* was positively regulated at 1 d in WL-S DH4, whereas in WL-T DH2, up-regulation occurred one day later, i.e., after 2 days of stress. In WL-T DH2, *lacs6* was also up-regulated in recovered plants (Rec) of both cucumber accessions (Figure 11E,F).

## 4. Discussion

Hypoxic stress in plants provokes changes on the morphological, physiological, and molecular levels. In this study, we compared responses to long-term waterlogging and recovery on the morphological and molecular levels in two cucumber accessions with divergent tolerance.

### 4.1. Morphological Changes in Response to Waterlogging Stress

In terms of morphological changes, discrepancies were observed between cucumber accessions. In WL-T DH2, no effect of hypoxic stress on plant height or the number of leaves was demonstrated, which in turn was observed in WL-S DH4, where hypoxic stress slowed down plant growth and reduced the number of leaves. In WL-T cucumbers, slower plant growth was first observed in the recovery period compared to control plants. The lack of difference in plant height in the WL-T accession in the context of hypoxia stress may be related to its tolerance. It has been shown that, in tolerant accessions, the height of stress-treated plants does not change compared to control plants, while in sensitive cucumber plants growth is slower [56,57]. Biomass accumulation in upper part of cucumber plants did not change under first waterlogging treatment in both DH lines. It was shown that under 8 days of waterlogging, biomass accumulation (DM:FM) was higher in treated cucumber plants, however the level of tolerance of tested cucumber was not provided. We carried out our analysis of a set of accessions with evidenced tolerance level [10], so based on our results, DM:FM has no impact on the tolerance level to hypoxia stress in tested lines. The second waterlogging (2xH) resulted in slower plant growth and biomass increase compared to the control plants, which may be due to the fact that, plants were focused on processes related to overcoming stress and not on the growth.

Differences in the response to stress were observed in the mass of the roots formed by cucumber plants. The increased ARN (hypocotyl-derived adventitious root number) that developed under hypoxic stress is considered to be a trait associated with tolerance and adaptation to limited oxygen access, as it facilitates gas exchange between the aerial zone and waterlogged soil [8,58,59]. Qi et al. [60] reported that tolerant and sensitive accessions were selected based on the ARN, where a large number of adventitious roots was observed in the tolerant accession, and a small number of AR was seen in the sensitive accession. These observations also correlate with the results we obtained, where WL-T DH2 produced a significantly greater mass of AR after 7 days of stress compared to the WL-S DH4. Enhanced development of ARN as an adaptation to waterlogging has also been observed in barley and tomato [56,61].

### 4.2. Changes at the Transcriptomic Level

The literature provides information on variations in the number of regulated transcripts under oxygen deprivation stress in genotypes with different levels of tolerance [13,62]. Kreuzwieser et al. [63] observed that the number of transcripts increased in the case of gray poplar genotypes characterized by increased hypoxia sensitivity. Here, the accession indicated as tolerant (DH2) demonstrated fewer transcripts with differential expression than the sensitive accession (DH4) under long-term waterlogging. The same trend was followed in cucumber by Xu et al. [17]; however, there the waterlogging stress lasted only 2 days. There are no transcriptomic data in the literature to compare cucumber accessions with contrasting responses to hypoxia. We also obtained a variable number of DEGs in plants that were treated again with hypoxic stress. In the case of the tolerant accession, the number of DEGs decreased after recurrent stress, while in the sensitive accession, the number of regulated transcripts increased. This may indicate that first waterlogging treatment enhanced tolerance to recurring waterlogging stress in the sensitive accession. An increased number of DEGs in primed plants, compared to non-primed plants, was also observed in rice, where salt shock was used as the priming factor [64] and in radish, where cold priming was applied [65]. To the best of our knowledge, there is no information on the differential number of regulated genes between plants treated once with hypoxic stress and plants treated again with stress, thus these results are novel and unique in the area of waterlogging tolerance in cucumber. Additionally, differences in the response to oxygen deprivation between DH2 and DH4 were observed at the transcriptomic level.

### 4.3. Effects of Waterlogging Stress Priming on the WL-T and WL-S Cucumber Accessions

Both cucumber accessions, under long-term hypoxic stress, enhanced pathways connected with translation i.e., ‘aminoacyl-tRNA biosynthesis’ and ‘RNA transport’, transcription i.e., ‘basal transcription factors’ and ‘spliceosome’, and signal transduction by activating ‘MAPK signaling pathway—plant’. In the WL tolerant cucumber, the ‘alanine, aspartate and glutamate metabolism’ pathway was activated, whereas in the WL sensitive cucumber, ‘biosynthesis of secondary metabolites’ was observed. The activation of ‘alanine, aspartate, and glutamate metabolism’ was also reported in cucumber hypocotyls of the WL-S cucumber accession [59]. Biosynthesis of secondary metabolites was also activated in the roots of rapeseed but, in contrast to our results, in the tolerant accession [13]. These differences could be explained manly due to the duration of exposure to waterlogging and the tissue that was analyzed. We can assume that cucumber plants cope with stress conditions by activating different pathways depending on exposure duration. There is no information on the use of RNA-Seq to find differences between accessions with contrasting hypoxia responses in cucumber at the root system level, so undoubtedly these results will provide sufficient knowledge in this area.

According to these results, in the WL tolerant accession after the second exposure to waterlogging, unique pathways were activated such as: ‘riboflavin metabolism’, ‘propanoate metabolism’, ‘zeatin biosynthesis’, and ‘valine, leucine, and isoleucine degradation’ related to amino acid metabolism. These pathways were also activated in the WL sensitive cucumber primed by 7 days waterlogging. To our knowledge, data associated with the response to a second treatment have not been reported so far. We can state that the pathway ‘valine, leucine, and isoleucine degradation’ is activated after a second exposure to waterlogging in cucumber. Additionally, in the WL sensitive accession, the ‘mRNA surveillance pathway’ was also activated in plants treated twice with waterlogging.

We also found differentially expressed genes in relation to control presenting contrasting expression levels with single and repeated waterlogging treatment. In the WL-T accession, the expression of *α-amylase/subtilisin inhibitor*, involved in starch and sucrose metabolism, was inhibited after a single waterlogging treatment, whereas after the second treatment, it was up-regulated. In barley, up-regulation of this gene has been reported in tolerant and moderately tolerant genotypes, but this observation was detected after 3 and 5 days of waterlogging. These contrasting results point out differences in the response to hypoxic stress between species [66]. In the WL-S cucumber, a similar expression pattern was demonstrated for *sugar transporter ERD6-like 16* and *calmodulin-like protein 8* genes. In radish, enhanced expression of the *sugar transporter ERD6-like* gene, considered to be one of the genes interacting with MaRAP2-4, provides abiotic stresses tolerance [67]. Since the WL-S accession revealed up-regulation of this gene after the second treatment (2xH), it could suggest that waterlogging acted as a priming factor in acquiring tolerance to waterlogging stress in the WL-S accession. Calmodulin-like proteins (CMLs) are sensors of Ca^2+^ and take part in calcium signaling [68]. It has been reported that CMLs may be involved in aerenchyma formation, allowing plants to perform gas exchange between roots and shoots [69].

### 4.4. Genes Potentially Involved in Tolerance to Long-Term Waterlogging in Cucumber

We indicated the genes potentially involved in acquiring tolerance to waterlogging stress in cucumber. These genes were uniquely up-regulated after a single waterlogging treatment in the WL-T accession and in the WL-S accession after the second exposure to stress. We observed strong up-regulation of *GDSL esterase/lipase* gene in our results. Enhanced expression of *GDSL esterase/lipase* gene and high accumulation of GDSL esterase/lipase protein were also observed in the tolerant cucumber line Zaoer-N in hypocotyls and roots, respectively [17,59]. These results suggest enhanced lipid catabolism in cucumbers with tolerance to hypoxic stress. As a result of *GDSL esterase/lipase* activity, glycerol and free fatty acids are produced. Glycerol may be used for carbon and energy supply and for adventitious roots development, as we confirmed here as being characteristic of the WL-T DH2 accession. Free fatty acids, due to the loss of cell walls during hypoxia, may provide components for newly generated cells.

Aspartate aminotransferase (AspAt) transfers N from aspartate to glutamate, which is a substrate of GABA synthesis, where GAD (glutamate decarboxylase) is involved [70]. Aspartate aminotransferase and glutamate decarboxylase also participate in amino acid metabolism and serves as regulators of cytoplasmic pH as it becomes lower during hypoxic stress, mostly due to enhanced lactic acid production [71]. In soybean hypocotyls, the *Aspartate aminotransferase* gene is up-regulated under flooding conditions [72]. Up-regulation of *AspAt* was also detected in the roots of a tolerant maize genotype [4]. In our study, up-regulation was demonstrated in the WL-T DH2 accession after a single waterlogging treatment, and as well as in the WL-S accession after subsequent stress induction. These results allow us to claim that the first stress treatment in the WL-S accession enhanced tolerance to oxygen deprivation.

The *glutamate decarboxylase* (*GAD*) gene is activated under oxygen deprivation and regulates the production of γ-amino butyric acid (GABA) [73]. GABA, by activating a number of genes, participates in plant adaptation to hypoxic stress caused by flooding [74]. Enhanced expression of *GAD*, exclusively in plants of the WL-T line cucumber after single hypoxia treatment and in plants of the WL-S line after the second treatment, may indicate that the WL-S cucumber line acquires tolerance to oxygen deprivation.

The *sucrose synthase* (*SuSy*) gene encodes an enzyme that participates in low oxygen level tolerance [75,76]. The role of *Susy* is the hydrolysis of sucrose to fructose and UDP-glucose in order to drive ATP production by glycolysis, which is important in oxygen deprivation tolerance [77]. Up-regulation of *SuSy* was determined in WL-tolerant cucumber [52], maize [78]), and barley [66] and it was also detected in our study, so it may be assumed that *SuSy* is involved in tolerance to long-term waterlogging.

In addition to the *SuSy* gene, we also detected up-regulation of *Triosephosphate isomerase*, *cytosolic* (*TPI*), an important glycolytic enzyme [79]. It has been reported that *TPI* is up-regulated in the roots of a WL-tolerant accession of maize [78], and our results confirm this tendency.

Expansins are responsible for alleviation of cell walls, resulting in its extension [80]. Increased expression has been observed under the influence of abiotic stress, such as drought [81], salinity [82], waterlogging [83,84], as well as also under the influence of low pH (~4.8). It was reported that increased development of root hair was observed in the RhEXPA4 transgenic line of *Arabidopsis* [85], which may correlate with the increased number of side roots that are produced under the limited oxygen access in tolerant plants. In maize, up-regulation of the *expansin-like A1* gene was found in the roots of a tolerant accession under waterlogging stress [84] as in our work. Higher expression of expansin genes contributes to the mitigation of hypoxic stress in root zones.

### 4.5. DEGs Determined in WL-S and WL-T Cucumbers in Recovery

The recovery period following the removal of hypoxic stress refers to reoxygenation [86]; however, the shift to normoxic conditions generates additional stresses, i.e., oxidative stress and dehydration due to damaged roots. For this reason, waterlogging is described as a sequential stress where the waterlogging and recovery periods pose distinct stressors. The ability to acclimate to both waterlogging and recovery is crucial for the determination of tolerance to hypoxic stress in plants [19,87]. The molecular mechanisms of the response to post-waterlogging reoxygenation in plants are poorly understood, and they have been investigated mainly within a short time frame after stress. In the recovery period, oxidative stress has been shown to be induced in plants because of dehydration caused by dysfunction of the root system and consequent generation of ROS, ABA, and ethylene [19,88]. Here, we exploited two cucumber accessions differing in waterlogging tolerance [10] and we compared the expression profiles of genes in plants after 14 days of recovery compared to control plants. Differential recovery between the WL-T DH2 and WL-S DH4 accessions was related to the activity of genes related to carbohydrate metabolic processes, which were significantly up-regulated in the WL-S accession. Among the pool of unique DEGs involved in carbohydrate metabolism in WL-S DH4 were the *probable pectate lyase 22*, *β-galactosidase 3*, and *endoglucanase 6* genes, encoding proteins involved in the degradation structural polysaccharides in plant cell walls. These genes were down-regulated under flooding stress, suggesting that the reduced accumulation of encoded proteins could lead to arrested root growth and the suppression of lateral root formation [89]. After 14 days of recovery, we observed increased expression of these genes only in the waterlogging sensitive cucumber, suggesting more intensive cell wall reconstruction processes and growth of the root system in comparison to WL-T cucumbers. The expression levels of the *ACC oxidase* 1 and *ACC oxidase* 3 genes, involved in the conversion of 1-aminocyclopropane-1-carboxylic acid (ACC) to ethylene, were also higher in the WL-S DH4 accession. These results suggest that long-term recovery in the WL-S cucumber was associated with ethylene biosynthesis at the transcriptional level. It has been reported that ethylene is synthesized during reoxygenation in submerged cucumber roots [90]. Tsai et al. [91] found that the expression levels of several *aminocyclopropane-1-carboxylic acid (ACC) synthase* and *ACC oxidase* genes were enhanced during the reoxygenation stage in *Arabidopsis*. They showed that ethylene plays a beneficial role in recovery by maintaining the balance of hormone signaling, including ethylene, as well as ABA and JA (Jasmonic acid). In WL-S DH4, the *naringenin 2-oxoglutarate 3-dioxygenase* gene, encoding an enzyme catalyzing the 3-hydroxylation of (2*S*)-flavanones such as naringenin to dihydroflavonols [92], was activated. Up-regulation of flavonoid pathway-related genes has been observed in potato, rice, and *Reaumuria soongorica* after exposure to drought and UV stress [93,94,95]; collectively, these studies indicate that the flavonoid pathway is involved in stress tolerance.

After 14 days of recovery, in the waterlogging tolerant cucumber (DH2), increased expression of *pyruvate dehydrogenase E1 component subunit α* gene was reported. Under aerobic conditions, pyruvate is converted to acetyl-CoA by the pyruvate dehydrogenase enzymatic complex. If oxygen levels decrease, NADH accumulates and the pyruvate dehydrogenase complex unit is inhibited [96]. Interestingly, in our study, the highest expression of this gene was found in recovery compared to control and only in the WL-tolerant cucumber accession.

### 4.6. qRT-PCR Assay Revealed Differences Throughout Waterlogging Stress Duration

Increased expression of *alcohol dehydrogenase* (*adh*) is used as a molecular marker for the stress response to hypoxia in plants [97]. Higher levels of *adh* transcripts were noted in the WL-T DH2 cucumber. Plants intensively activate the alcoholic fermentation pathway in order to produce NAD^+^ and maintain glycolysis under conditions of low oxygen concentration. NAD^+^ regeneration from NADH is considered to be the most important function of the alcoholic fermentation pathway under oxygen restricted conditions [98]. An increased expression level of *adh* under hypoxic stress was also demonstrated in the roots of radish [99], poplar [63], and the cucumber genotype Zaoer, which has a high level of tolerance to flooding stress [52]. Relative expression levels of *adh* obtained for both cucumber accessions by qRT-PCR correspond to RNA-Seq results.

Ethylene is a plant hormone known as a signaling and regulatory molecule in response to plant hypoxic stress [100]. It is synthetized from methionine, which is first converted to S-adenosylmethionine (AdoMet) by S-adenosylmethionine synthase, then converted to 1-aminocyclopropane-1-carboxylate (ACC) by ACC synthase (ACS) [101]. Ethylene synthesis requires oxygen at the level of 1-aminocyclopropane-1-carboxylate oxidase (*aco*), which catalyzes the final step in ethylene biosynthesis. Differences in the expression level of the *aco* gene in response to hypoxic stress were found in both cucumber accessions, demonstrating contrasting reactions to limited oxygen access, which indicates the different mechanisms related to the response to oxygen deprivation in the cucumber root system. Increased expression of the *aco* gene under hypoxic stress was also demonstrated by Qi et al. [52] in cucumber roots 4 hours after stress induction. *Aco* expression level determined by qRT-PCR in WL-S DH4 correlated with RNA-Seq results.

Long-chain acyl-CoA synthases (LACS) activate free fatty acids to acyl-CoA thioesters. This class of enzymes is involved in several fatty acid metabolic pathways, including phospholipid biosynthesis and β-fatty acid oxidation [102], which are essential for maintaining cell homeostasis [103]. Lipids are essential cellular components that not only provide the structural basis for cell membranes and energy for metabolic processes, but also serve as signals in plant responses to environmental signals [104]. In roots, the LACS gene product may be involved in the suberin and cuticle waxes biosynthesis [105]. The cutcle can change in response to various abiotic or biotic stresses [106]. It was found that the relative expression level of the *lacs 6* gene was significantly higher in the DH2 cucumber line, indicative of tolerance to hypoxic stress, which may suggest that the β-lipid oxidation process was more intense. This also correlates with the amount of ascorbic acid and glutathione determined in the tested cucumber lines (unpublished data), which are components of the antioxidant system in plants, protecting cells against the negative effects of reactive oxygen species (ROS). qRT-PCR revealed higher expression of *lacs6* in WL-S after 1xH and 2xH treatment than it was determined in the RNA-Seq approach, whereas in WL-S, results obtained by qRT-PCR correlated with RNA-Seq.

## 5. Conclusions

Nowadays, developing methods to improve tolerance to abiotic and biotic stresses is the main goal of breeders to reduce losses associated with climate change [107] in the context of food protection [31]. In this study, we obtained results supporting waterlogging as a priming factor against waterlogging stress in cucumber. The results obtained in this study provide new and unique information regarding the responses to long-term hypoxia in cucumber in terms of priming. However, a significant percentage of the identified DEGs were functionally unannotated and uncharacterized, but can be assigned to specific cucumber genes, transposable elements, or ncRNA that may be involved in post-transcriptional regulation. This aspect requires further investigation and will be examined in the future.

## Figures and Tables

**Table 1:** Summary of de novo transcriptome data assembly of *Cucumis sativus* L.

	Trascripts
Total numer (bp)	35,712
Total length (bp)	79,877,390
N50 length (bp)	2711
Minimum length (bp)	162
Maximum length (bp)	20,773
Average length (bp)	2236

**Table 2:** Numbers of differentially expressed genes (DEGs).

Regulation	WL-T DH2				WL-S DH4			
	1xH vs. Ctrl	Rec vs. Ctrl	2xH vs. Ctrl	1xH vs. 2xH	1xH vs. Ctrl	Rec vs. Ctrl	2xH vs. Ctrl	1xH vs. 2xH
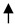	2584 (493) *	355 (66)	2310 (474)	78 (13)	4211 (948)	892 (164)	5453 (1120)	1649 (355)
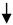	3373 (560)	299 (51)	2697 (473)	348 (71)	4716 (809)	985 (249)	6166 (1112)	1196 (238)
Total	5957 (1053)	654 (117)	5007 (947)	426 (84)	8927 (1757)	1,877 (413)	11,619 (2232)	2845 (593)

* Number of differentially expressed genes for which FDR < 0.05. Numbers in brackets correspond to number of functionally uncharacterized and unannotated.

## Data Availability

Data is contained within the article or supplementary material. The RNA-Seq datasets generated for this study are deposited in the NCBI under BioProject PRJNA678740 and can be found in the GenBank Short Read Archive (SRA) under Acc. No. SSR13083584 to SSR13083607.

## Supplementary Materials

The following are available online at https://www.mdpi.com/2073-4425/12/2/189/s1, Figure S1: Number of Differentially Expressed Genes identified by DESeq2 and edgeR in two cucumber DH lines under waterlogging stress, Figure S2: Pictorial representation of genes in WL-T DH2 and WL-S DH4 accessions associated with various MapMan functional metabolism categories under waterlogging stress, Figure S3: Pictorial representation of genes in WL-T DH2 (A, B) and WL-S DH4 (C, D) accessions associated with MapMan functional regulation overview under single (1xH) and repeated (2xH) waterlogging stress. Table S1: Genes data and primers used in the analysis, Table S2: Summary of RNA-Seq and mapping details, Table S3: Statistics of annotation results for *Cucumis sativus* L. transcripts, Table S4: Genes with opposite expression in cucumber plants treated once (1xH) and twice (2xH) with waterlogging, Table S5: Gene ontology enrichment of DEGs identified in WL-T in Rec, Table S6: Gene ontology enrichment of DEGs identified in WL-S in Rec, Data S1: KEGG mapping of *Cucumis sativus* L. transcripts, Data S2: DEGs identified in comparison DH2 1xH vs. Ctrl, Data S3: DEGs identified in comparison DH2 Rec vs. Ctrl, Data S4: DEGs identified in comparison DH2 2xH vs. Ctrl, Data S5: DEGs identified in comparison DH2 1xH vs. 2xH, Data S6: DEGs identified in comparison DH4 1xH vs. Ctrl, Data S7: DEGs identified in comparison DH4 Rec vs. Ctrl, Data S8: DEGs identified in comparison DH4 2xH vs. Ctrl, Data S9: DEGs identified in comparison DH4 1xH vs. 2xH, Data S10: Common 305 DEGs identified in DH2 1xH vs. Ctrl and DH4 2xH vs. Ctrl.
