# Supplementary material for: Long-Term Waterlogging as Factor Contributing to Hypoxia Stress Tolerance Enhancement in Cucumber: Comparative Transcriptome Analysis of Waterlogging Sensitive and Tolerant Accessions
Source: Genes (Basel). 2021 Jan 28;12(2):189. doi: 10.3390/genes12020189 (PMC7912563; doi:10.3390/genes12020189)

Supplementry Materials Figure S1. Number of Differentially Expressed Genes identified by DESeq2 and edgeR in two cucumber DH lines under waterlogging stress

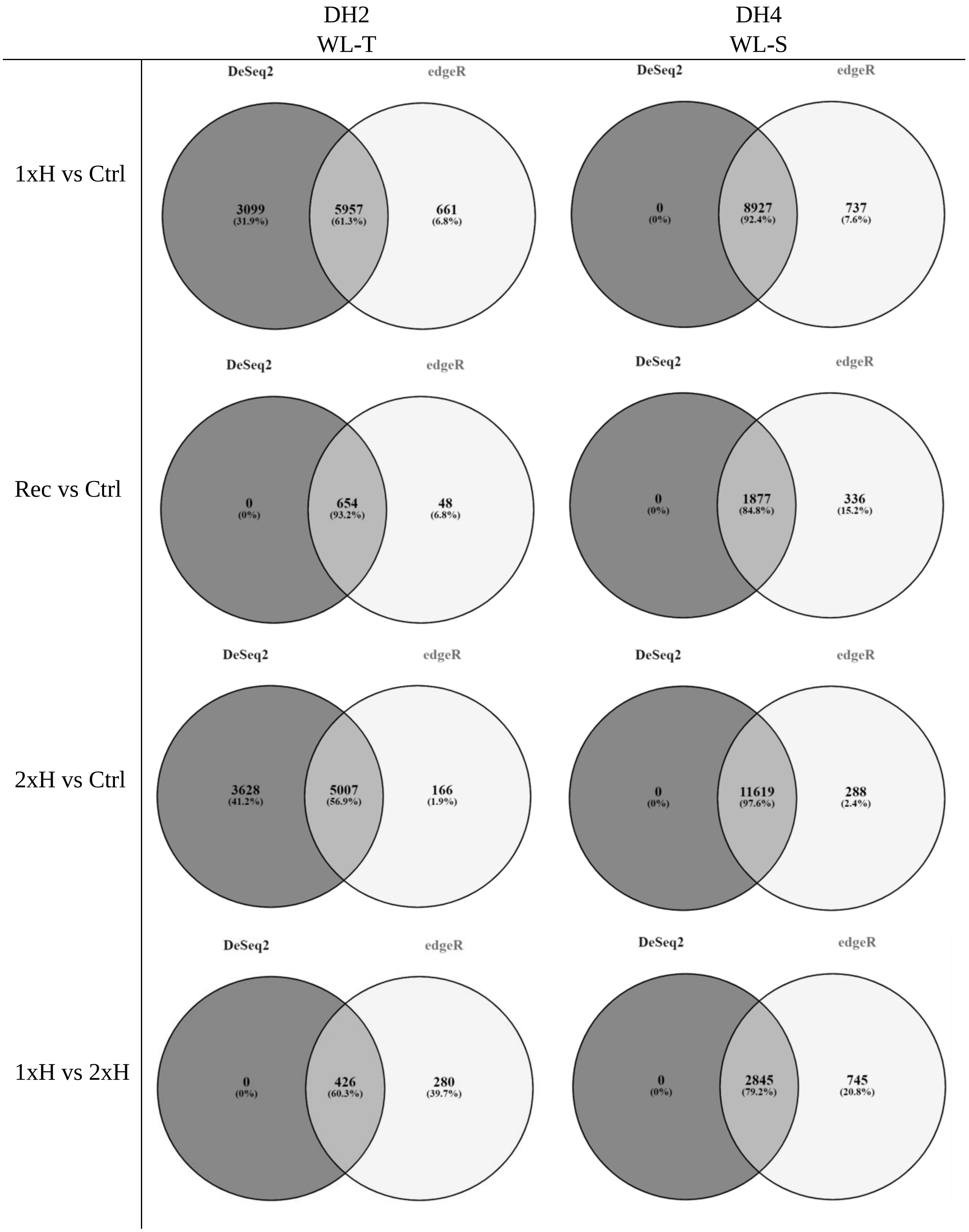

Supplementary Materials Figure S2. Pictorial representation of genes in WL-T DH2 and WL-S DH4 accessions associated with various MapMan functional metabolism categories under waterlogging stress.

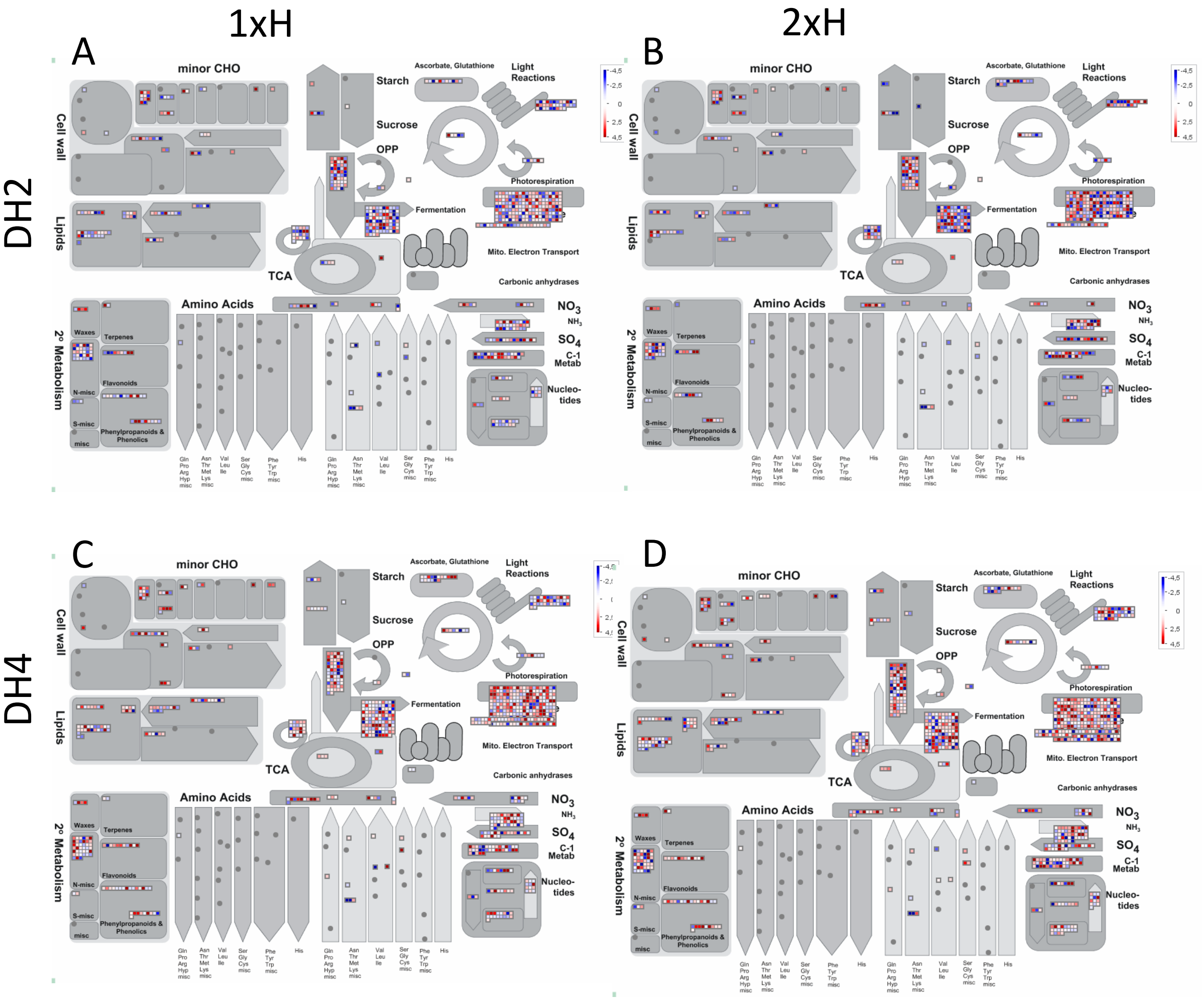

Supplementary Materials Figure S3. Pictorial representation of genes in WL-T DH2 (A, B) and WL-S DH4 (C, D) accessions associated with MapMan functional regulation overview under single (1xH) and repeated (2xH) waterlogging stress.

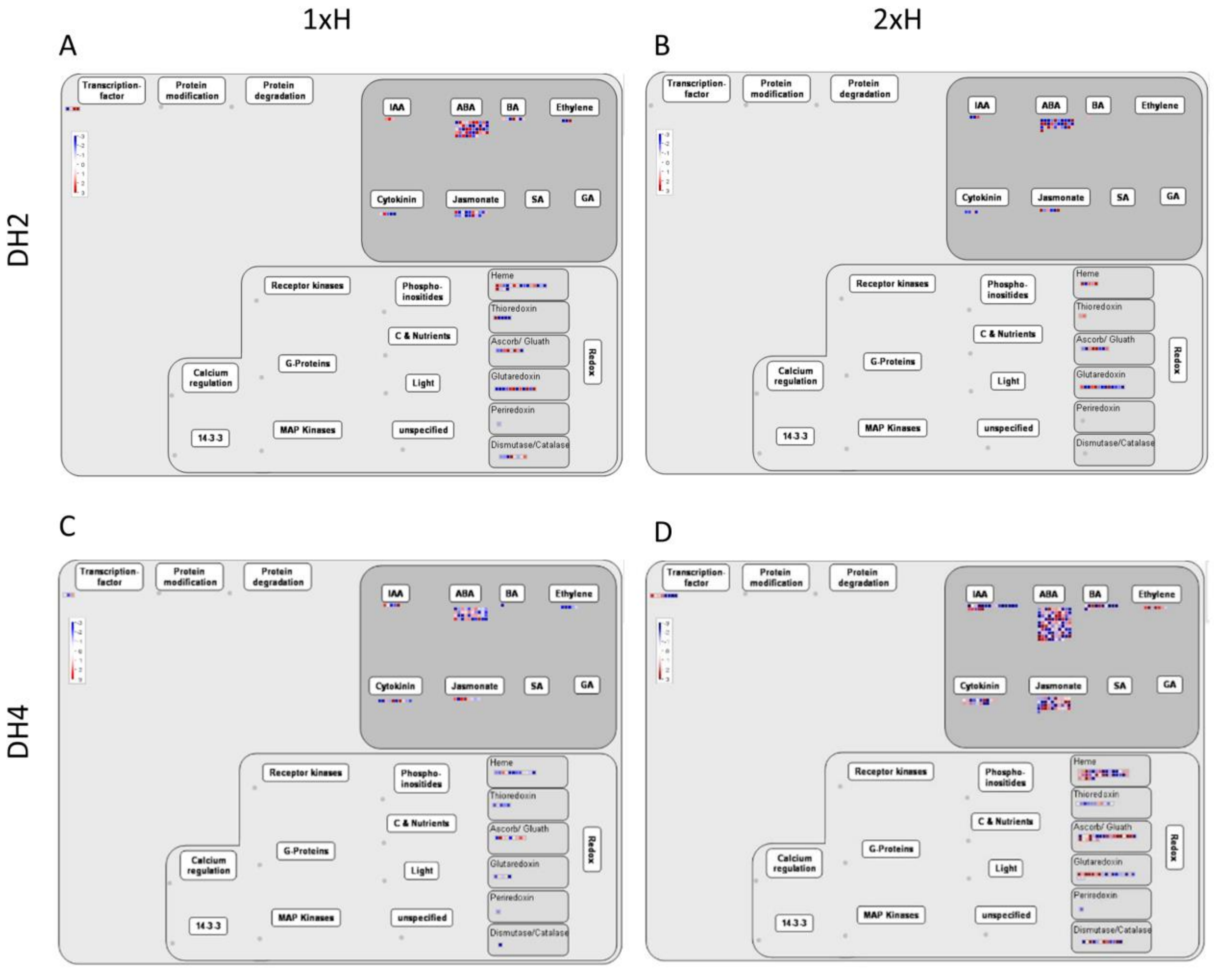

Supplement: Supplementary file 1 [file genes-12-00189-s001.zip › genes-1035154-supplementary_/Supplementary Materials Figures S1-S3.pdf]
