# Supplementary material for: Long-Term Waterlogging as Factor Contributing to Hypoxia Stress Tolerance Enhancement in Cucumber: Comparative Transcriptome Analysis of Waterlogging Sensitive and Tolerant Accessions
Source: Genes (Basel). 2021 Jan 28;12(2):189. doi: 10.3390/genes12020189 (PMC7912563; doi:10.3390/genes12020189)
Supplement: Supplementary file 1 [file genes-12-00189-s001.zip › genes-1035154-supplementary_/Supplementary Materials Tables S1-S6.docx]

**Supplementary Materials Table S1.** Genes data and primers used in the analyses.

| Symbol | Gene | Source | Primer sequence (5’ - 3’) | Product size (bp) |
| --- | --- | --- | --- | --- |
| *act* | actin | Qi et al. 2012 | TGGACTCTGGTGATGGTGTTA | 150 |
|  |  |  | CAATGAGGGATGGTGGAAAA |  |
| *tua* | tubulin alpha chain | Wang et al. 2014 | ACGCTGTTGGTGGTGGTAC | 210 |
|  |  |  | GAGAGGGGTAAACAGTGAATC |  |
| *adh* | alcohol dehydrogenase | Xuewen et al. 2014 | AGACAGCCAACATGAACAAC | 139 |
|  |  |  | ATCTTCTCCGAATCAACACT |  |
| *aco* | 1-aminocyclopropane-1-carboxylate oxidase | XM_004141001.2 | GGCTTCTTTGTAGTAGAGAACCAT | 75 |
|  |  |  | ATGTTTATTTACCAACAGCTTCACC |  |
| *lacs6* | long chain acyl-CoA synthetase 6 | XM_004134094.2 | TGCGAAGTGCAAGTTTGTCG | 164 |
|  |  |  | TTGCTCTCGGGTCATTGCAG |  |

**Supplementary Materials Table S2.** Summary of RNA-Seq and mapping details.

| Accession | Treatment | Biological replicate | Total reads | Raw base (Gbp) | Clean reads | Clean base (Gbp) | GC content  (%) | Total mapped to cucumber genome | Uniquely mapped | Multiple mapped |
| --- | --- | --- | --- | --- | --- | --- | --- | --- | --- | --- |
| DH2  WL-T | Ctrl | 1 | 52,149,594 | 5.2 | 46,870,012 (89.88%) | 4.6 | 43 | 23,435,003 (50.00%) | 22,624,469 | 456,740 |
|  |  | 2 | 47,232,946 | 4.7 | 42,232,426 (89.41%) | 4.1 | 43 | 21,116,211 (50.00%) | 20,244,752 | 424,124 |
|  |  | 3 | 44,810,380 | 4.5 | 39,977,348 (89.21%) | 3.9 | 43 | 19,988,668 (50.00%) | 19,255,089 | 378,035 |
|  | 1xH | 1 | 59,515,066 | 5.9 | 53,037,022 (89.12%) | 5.2 | 43 | 26,518,410 (50.00%) | 25,135,274 | 668,789 |
|  |  | 2 | 61,925,418 | 6.2 | 55,025,194 (88.86%) | 5.4 | 43 | 27,512,591 (50.00%) | 26,354,066 | 557,198 |
|  |  | 3 | 50,836,202 | 5.1 | 45,040,650 (88.60%) | 4.4 | 43 | 22,520,316 (50.00%) | 21,565,688 | 456,973 |
|  | Rec | 1 | 51,771,498 | 5.2 | 46,633,024 (90.07%) | 4.6 | 43 | 23,316,502 (50.00%) | 22,090,051 | 550,156 |
|  |  | 2 | 55,081,386 | 5.5 | 48,903,790 (88.78%) | 4.8 | 43 | 24,451,869 (50.00%) | 23,349,963 | 596,070 |
|  |  | 3 | 38,725,334 | 3.9 | 34,440,028 (88.93%) | 3.4 | 43 | 17,220,004 (50.00%) | 16,310,144 | 414,671 |
|  | 2xH | 1 | 40,067,176 | 4.0 | 35,312,134 (88.13%) | 3.4 | 43 | 17,656,047 (50.00%) | 16,553,659 | 475,612 |
|  |  | 2 | 40,603,530 | 4.0 | 36,060,886 (88.81%) | 3.5 | 44 | 18,030,428 (50.00%) | 16,528,323 | 501,616 |
|  |  | 3 | 41,516,084 | 4.1 | 36,841,994 (88.74%) | 3.6 | 44 | 18,420,985 (50.00%) | 17,174,108 | 481,426 |
| DH4  WL-S | Ctrl | 1 | 46,908,784 | 4.7 | 41,671,718 (88.84%) | 4.1 | 43 | 20,835,849 (50.00%) | 20,070,855 | 428,433 |
|  |  | 2 | 39,366,456 | 3.9 | 35,257,188 (89.56%) | 3.4 | 43 | 17,628,577 (50.00%) | 16,970,524 | 354,944 |
|  |  | 3 | 37,545,980 | 3.7 | 33,434,730 (89.05%) | 3.3 | 43 | 16,717,363 (50.00%) | 16,003,543 | 331,090 |
|  | 1xH | 1 | 42,958,076 | 4.2 | 37,849,698 (88.11%) | 3.7 | 43 | 18,924,841 (50.00%) | 17,933,705 | 449,832 |
|  |  | 2 | 49,792,990 | 5.0 | 44,168,950 (88.71%) | 4.3 | 42 | 22,084,466 (50.00%) | 20,715,416 | 464,787 |
|  |  | 3 | 42,058,784 | 4.2 | 36,957,082 (87.87%) | 3.6 | 43 | 18,478,528 (50.00%) | 17,472,446 | 419,057 |
|  | Rec | 1 | 46,982,862 | 4.9 | 40,876,592 (87.00%) | 4.0 | 43 | 20,438,294 (50.00%) | 19,540,348 | 484,488 |
|  |  | 2 | 55,591,228 | 5.5 | 49,025,446 (88.19%) | 4.8 | 43 | 24,512,717 (50.00%) | 23,335,561 | 634,030 |
|  |  | 3 | 55,234,324 | 5.5 | 48,667,734 (88.11%) | 4.8 | 43 | 24,333,867 (50.00%) | 23,314,200 | 555,632 |
|  | 2xH | 1 | 54,929,822 | 5.5 | 48,881,366 (88.99%) | 4.8 | 43 | 24,440,681 (50.00%) | 23,001,879 | 729,715 |
|  |  | 2 | 48,136,774 | 4.8 | 42,515,466 (88.32%) | 4.2 | 43 | 21,257,732 (50.00%) | 19,985,257 | 505,890 |
|  |  | 3 | 52,686,956 | 5.3 | 46,501,536 (88.26%) | 4.5 | 44 | 23,250,729 (50.00%) | 21,286,518 | 750,340 |
|  |  | Average | 48,184,485 | 4.8 | 42,757,583 | 4.2 |  |  |  |  |
|  |  | Total | 1,156,427,650 | 115.2 | 1,026,182,014 | 100.3 |  |  |  |  |

**Supplementary Materials Table S3**. Statistics of annotation results for *Cucumis sativus* L. transcripts.

| Database | Number of annotated transcripts | Percentage (%) |
| --- | --- | --- |
| sprot_Top_BLASTX_hit | 29,077 | 81.42 |
| sprot_Top_BLASTP_hit | 26,883 | 75.28 |
| Pfam | 26,082 | 73.03 |
| SignalP | 2,285 | 6.40 |
| TmHMM | 6,924 | 19.39 |
| EggNOG | 24,509 | 68.63 |
| KEGG | 25,590 | 71.66 |
| GO | 27,537 | 77.11 |
| At least in one database | 15,623 |  |

**Supplementary Materials Table S4.** Genes with opposite expression in cucumber plants treated once (1xH) and twice (2xH) with waterlogging.

| **Table 2.** | Description/Annotation | Log_2_FC | | GO terms* |
| --- | --- | --- | --- | --- |
|  |  | 1xH vs Ctrl | 2xH vs Ctrl |  |
| **WL-T DH2:** | | | | |
| **XLOC_003864** | Alpha-amylase/subtilisin inhibitor | -2.96 | 2.58 | MF: GO:0015066 alpha-amylase inhibitor activity |
| XLOC_006633 | Beta-amyrin 11-oxidase | -1.37 | 1.54 | MF: GO:0020037 heme binding |
| XLOC_009457 | Probable isoaspartyl peptidase/L-asparaginase 2 | -1.30 | 1.68 | MF: GO:0004067 asparaginase activity |
| **WL-S DH4:** | | | | |
| XLOC_017095 | Unknown | -2.46 | 3.11 | . |
| XLOC_014798 | Sugar transporter ERD6-like 16 | -2.16 | 2.05 | BP: GO:0035428 hexose transmembrane transport |
| XLOC_002728 | Calmodulin-like protein 8 | -1.62 | 1.75 | BP: GO:0019722 calcium-mediated signaling  BP: GO:0005513 detection of calcium ion |
| XLOC_010098 | Unknown | -1.41 | 1.21 | CC: GO:0016021 integral component of membrane |
| XLOC_000765 | Protein SMAX1-LIKE 4 | -1.01 | 1.14 | BP: GO:0033500 carbohydrate homeostasis BP: GO:0010233 phloem transport BP: GO:0019538 protein metabolic process BP: GO:0006355 regulation of transcription, DNA-templated |
| XLOC_000416 | Unknown | 1.01 | -4.11 | . |
| XLOC_008208 | Unknown | 1.04 | -2.44 | . |
| XLOC_005736 | Unknown | 1.05 | -1.92 | . |
| XLOC_009831 | 1-aminocyclopropane-1-carboxylate synthase | 1.12 | -1.48 | MF: GO:0016847 1-aminocyclopropane-1-carboxylate synthase activity MF: GO:0030170 pyridoxal phosphate binding BP: GO:0009693 ethylene biosynthetic process` |
| XLOC_011509 | Protein JINGUBANG | 1.20 | -1.71 | BP: GO:0009846 pollen germination |
| XLOC_013197 | Unknown | 1.21 | -1.16 | . |
| XLOC_012101 | Heavy metal-associated isoprenylated plant protein 39 | 1.22 | -1.58 | BP: GO:0046916 cellular transition metal ion homeostasis BP: GO:0030001 metal ion transport |
| XLOC_012888 | Probable ribose-5-phosphate isomerase 2 | 1.24 | -2.72 | BP: GO:0008219 cell death BP: GO:0009052 pentose-phosphate shunt, non-oxidative branch |
| XLOC_003380 | Transcriptionfactor MYB44 | 1.28 | -2.67 | BP: GO:0009738 abscisic acid-activated signaling pathway BP: GO:0030154 cell differentiation BP: GO:2000022 regulation of jasmonic acid mediated signaling pathway BP: GO:2000031 regulation of salicylic acid mediated signaling pathway BP: GO:0009737 response to abscisic acid BP: GO:0009733 response to auxin BP: GO:0046686 response to cadmium ion BP: GO:0010200 response to chitin BP: GO:0009723 response to ethylene BP: GO:0009739 response to gibberellin BP: GO:0009753 response to jasmonic acid BP: GO:0009751 response to salicylic acid BP: GO:0009414 response to water deprivation |
| XLOC_002592 | Serine/threonine-protein kinase RIPK | 1.30 | -2.19 | BP: GO:0007166 cell surface receptor signaling pathway BP: GO:0006468 protein phosphorylation |
| XLOC_012321 | Putative serine/threonine-protein kinase-like protein CCR3 | 1.52 | -2.27 | MF: GO:0005524 ATP binding MF: GO:0004674 protein serine/threonine kinase activity |
| XLOC_000742 | Unknown | 1.58 | -1.96 | . |
| XLOC_017116 | S-type anion channel SLAH1 | 1.60 | -1.77 | BP: GO:0006873 cellular ion homeostasis BP: GO:1901529 positive regulation of anion channel activity BP: GO:0009414 response to water deprivation |
| XLOC_011085 | Unknown | 1.70 | -2.28 | . |
| XLOC_010908 | Ethylene-responsive transcription factor ERF026 | 1.71 | -2.59 | BP GO:0009873 ethylene-activated signaling pathway BP: GO:0006351 transcription, DNA-templated |
| XLOC_009715 | IN2-2 protein | 1.82 | -1.43 | . |
| XLOC_016466 | Carbamoyl-phosphate synthase large chain, chloroplastic | 1.88 | -1.89 | BP: GO:0006526 arginine biosynthetic process BP: GO:0016036 cellular response to phosphate starvation BP: GO:0000050 urea cycle |
| XLOC_008177 | AAA-ATPase ASD, mitochondrial | 1.96 | -1.18 | BP: GO:0009737 response to abscisic acid BP: GO:0009414 response to water deprivation |
| XLOC_006223 | Unknown | 1.97 | -1.52 | . |
| XLOC_007688 | Ninja-family protein AFP3 | 2.01 | -2.07 | BP: GO:0007165 signal transduction |
| XLOC_004238 | Unknown | 2.02 | -1.71 | . |
| XLOC_003800 | Probable glycosyl transferase At3g42180 | 2.02 | -1.65 | BP: GO:0071555 cell wall organization BP: GO:0006486 protein glycosylation |
| XLOC_011616 | Unknown | 2.15 | -1.98 | . |
| XLOC_011942 | MLP-like protein 328 | 2.23 | -1.36 | BP: GO:0009735 response to cytokinin |
| XLOC_014985 | Calcium-binding protein PBP1 | 2.42 | -1.88 | MF: GO:0005509 calcium ion binding BP: GO:0009733 response to auxin |
| XLOC_003263 | Unknown | 2.40 | -5.09 | . |
| XLOC_015719 | Unknown | 2.75 | -1.75 | . |
| XLOC_014021 | Cinnamoyl-CoAreductase1 | 2.83 | -2.17 | BP: GO:0006952 defense response BP: GO:0009809 lignin biosynthetic process |
| XLOC_005987 | Unknown | 3.41 | -2.73 | . |

**Supplementary Materials Table S5.** Gene ontology enrichment of DEGs identified in WL-T in Rec.

| **up-regulated** | | | | | | | |  | **down-regulated** | | | | | | |
| --- | --- | --- | --- | --- | --- | --- | --- | --- | --- | --- | --- | --- | --- | --- | --- |
| **BP** | GO.ID | Term | Annotated | Significant | Expected | classicFisher | qvalue |  | GO.ID | Term | Annotated | Significant | Expected | classicFisher | qvalue |
|  | GO:0042737 | drug catabolic process | 196 | 30 | 4.29 | 2.60E-17 | 3.12E-15 |  | GO:0006721 | terpenoid metabolic process | 132 | 20 | 2.63 | 1.40E-12 | 1.68E-10 |
|  | GO:0055114 | oxidation-reduction process | 990 | 65 | 21.66 | 2.70E-16 | 1.62E-14 |  | GO:0016114 | terpenoid biosynthetic process | 119 | 18 | 2.37 | 1.90E-11 | 1.14E-09 |
|  | GO:0017001 | antibiotic catabolic process | 87 | 20 | 1.9 | 2.00E-15 | 7.99E-14 |  | GO:0006720 | isoprenoid metabolic process | 169 | 20 | 3.36 | 1.40E-10 | 5.59E-09 |
|  | GO:0042744 | hydrogen peroxide catabolic process | 77 | 18 | 1.68 | 3.80E-14 | 1.01E-12 |  | GO:0055085 | transmembrane transport | 616 | 37 | 12.25 | 1.40E-09 | 4.07E-08 |
|  | GO:0042743 | hydrogen peroxide metabolic process | 101 | 20 | 2.21 | 4.20E-14 | 1.01E-12 |  | GO:0008299 | isoprenoid biosynthetic process | 155 | 18 | 3.08 | 1.70E-09 | 4.07E-08 |
|  | GO:0051187 | cofactor catabolic process | 107 | 19 | 2.34 | 1.50E-12 | 3.00E-11 |  | GO:0015807 | L-amino acid transport | 10 | 6 | 0.2 | 1.10E-08 | 2.20E-07 |
|  | GO:0072593 | reactive oxygen species metabolic proces... | 154 | 21 | 3.37 | 1.90E-11 | 3.25E-10 |  | GO:0001504 | neurotransmitter uptake | 7 | 5 | 0.14 | 6.10E-08 | 7.31E-07 |
|  | GO:0016999 | antibiotic metabolic process | 175 | 22 | 3.83 | 3.20E-11 | 4.79E-10 |  | GO:0051938 | L-glutamate import | 7 | 5 | 0.14 | 6.10E-08 | 7.31E-07 |
|  | GO:0017144 | drug metabolic process | 594 | 39 | 12.99 | 6.20E-10 | 8.26E-09 |  | GO:0089718 | amino acid import across plasma membrane | 7 | 5 | 0.14 | 6.10E-08 | 7.31E-07 |
|  | GO:0006950 | response to stress | 2583 | 98 | 56.51 | 2.70E-09 | 3.24E-08 |  | GO:0098712 | L-glutamate import across plasma membran... | 7 | 5 | 0.14 | 6.10E-08 | 7.31E-07 |
|  | GO:0006979 | response to oxidative stress | 303 | 25 | 6.63 | 1.20E-08 | 1.31E-07 |  | GO:0043090 | amino acid import | 14 | 6 | 0.28 | 1.50E-07 | 1.37E-06 |
|  | GO:0009607 | response to biotic stimulus | 791 | 41 | 17.3 | 1.90E-07 | 1.90E-06 |  | GO:0006811 | ion transport | 581 | 32 | 11.55 | 1.50E-07 | 1.37E-06 |
|  | GO:0006952 | defense response | 1065 | 48 | 23.3 | 9.80E-07 | 9.03E-06 |  | GO:0015813 | L-glutamate transmembrane transport | 8 | 5 | 0.16 | 1.60E-07 | 1.37E-06 |
|  | GO:0050896 | response to stimulus | 4341 | 131 | 94.96 | 3.10E-06 | 2.65E-05 |  | GO:1902475 | L-alpha-amino acid transmembrane transpo... | 8 | 5 | 0.16 | 1.60E-07 | 1.37E-06 |
|  | GO:0000272 | polysaccharide catabolic process | 129 | 13 | 2.82 | 4.80E-06 | 3.84E-05 |  | GO:0098739 | import across plasma membrane | 16 | 6 | 0.32 | 3.90E-07 | 3.12E-06 |
|  | GO:0006030 | chitin metabolic process | 26 | 6 | 0.57 | 1.60E-05 | 9.59E-05 |  | GO:0055114 | oxidation-reduction process | 990 | 43 | 19.69 | 7.80E-07 | 5.84E-06 |
|  | GO:0006032 | chitin catabolic process | 26 | 6 | 0.57 | 1.60E-05 | 9.59E-05 |  | GO:0042445 | hormone metabolic process | 217 | 17 | 4.32 | 1.50E-06 | 1.06E-05 |
|  | GO:0046348 | amino sugar catabolic process | 26 | 6 | 0.57 | 1.60E-05 | 9.59E-05 |  | GO:0015800 | acidic amino acid transport | 12 | 5 | 0.24 | 2.10E-06 | 1.40E-05 |
|  | GO:1901071 | glucosamine-containing compound metaboli... | 26 | 6 | 0.57 | 1.60E-05 | 9.59E-05 |  | GO:0006865 | amino acid transport | 76 | 10 | 1.51 | 2.40E-06 | 1.51E-05 |
|  | GO:1901072 | glucosamine-containing compound cataboli... | 26 | 6 | 0.57 | 1.60E-05 | 9.59E-05 |  | GO:0015849 | organic acid transport | 120 | 12 | 2.39 | 4.60E-06 | 2.63E-05 |
|  | GO:0051707 | response to other organism | 723 | 34 | 15.82 | 1.90E-05 | 0.000108 |  | GO:0046942 | carboxylic acid transport | 120 | 12 | 2.39 | 4.60E-06 | 2.63E-05 |
|  | GO:0006026 | aminoglycan catabolic process | 27 | 6 | 0.59 | 2.10E-05 | 0.000114 |  | GO:0009813 | flavonoid biosynthetic process | 67 | 9 | 1.33 | 6.60E-06 | 3.60E-05 |
|  | GO:0043207 | response to external biotic stimulus | 730 | 34 | 15.97 | 2.40E-05 | 0.000125 |  | GO:0006714 | sesquiterpenoid metabolic process | 37 | 7 | 0.74 | 7.00E-06 | 3.65E-05 |
|  | GO:0051186 | cofactor metabolic process | 494 | 26 | 10.81 | 3.10E-05 | 0.000155 |  | GO:0065008 | regulation of biological quality | 971 | 40 | 19.31 | 7.40E-06 | 3.70E-05 |
|  | GO:0098542 | defense response to other organism | 562 | 28 | 12.29 | 4.10E-05 | 0.000197 |  | GO:0006836 | neurotransmitter transport | 16 | 5 | 0.32 | 1.10E-05 | 5.27E-05 |
|  | GO:0009605 | response to external stimulus | 1026 | 42 | 22.44 | 5.30E-05 | 0.000244 |  | GO:0006820 | anion transport | 253 | 17 | 5.03 | 1.20E-05 | 5.53E-05 |
|  | GO:0016052 | carbohydrate catabolic process | 212 | 15 | 4.64 | 6.50E-05 | 0.00026 |  | GO:0080144 | amino acid homeostasis | 17 | 5 | 0.34 | 1.50E-05 | 6.66E-05 |
|  | GO:0001906 | cell killing | 11 | 4 | 0.24 | 6.50E-05 | 0.00026 |  | GO:0010817 | regulation of hormone levels | 289 | 18 | 5.75 | 1.90E-05 | 8.13E-05 |
|  | GO:0031640 | killing of cells of other organism | 11 | 4 | 0.24 | 6.50E-05 | 0.00026 |  | GO:0055081 | anion homeostasis | 43 | 7 | 0.86 | 2.00E-05 | 8.27E-05 |
|  | GO:0044364 | disruption of cells of other organism | 11 | 4 | 0.24 | 6.50E-05 | 0.00026 |  | GO:0042446 | hormone biosynthetic process | 163 | 13 | 3.24 | 2.20E-05 | 8.79E-05 |
| **MF** | GO:0004866 | endopeptidase inhibitor activity | 41 | 18 | 0.98 | 4.70E-19 | 2.82E-17 |  | GO:0016705 | oxidoreductase activity, acting on paire... | 235 | 23 | 4.6 | 2.00E-10 | 1.04E-08 |
|  | GO:0030414 | peptidase inhibitor activity | 41 | 18 | 0.98 | 4.70E-19 | 2.82E-17 |  | GO:0022857 | transmembrane transporter activity | 796 | 44 | 15.58 | 2.60E-10 | 1.04E-08 |
|  | GO:0061135 | endopeptidase regulator activity | 42 | 18 | 1 | 8.00E-19 | 3.20E-17 |  | GO:0005506 | iron ion binding | 197 | 21 | 3.86 | 2.60E-10 | 1.04E-08 |
|  | GO:0061134 | peptidase regulator activity | 43 | 18 | 1.03 | 1.30E-18 | 3.90E-17 |  | GO:0005215 | transporter activity | 950 | 47 | 18.6 | 2.20E-09 | 6.59E-08 |
|  | GO:0016491 | oxidoreductase activity | 1207 | 73 | 28.83 | 3.10E-14 | 7.43E-13 |  | GO:0004497 | monooxygenase activity | 186 | 18 | 3.64 | 2.40E-08 | 5.75E-07 |
|  | GO:0020037 | heme binding | 252 | 29 | 6.02 | 1.80E-12 | 3.60E-11 |  | GO:0015318 | inorganic molecular entity transmembrane... | 469 | 29 | 9.18 | 3.80E-08 | 7.59E-07 |
|  | GO:0004857 | enzyme inhibitor activity | 121 | 20 | 2.89 | 7.60E-12 | 1.30E-10 |  | GO:0015186 | L-glutamine transmembrane transporter ac... | 7 | 5 | 0.14 | 5.60E-08 | 9.59E-07 |
|  | GO:0048037 | cofactor binding | 858 | 55 | 20.49 | 1.00E-11 | 1.46E-10 |  | GO:0015075 | ion transmembrane transporter activity | 482 | 29 | 9.43 | 6.90E-08 | 1.03E-06 |
|  | GO:0046906 | tetrapyrrole binding | 271 | 29 | 6.47 | 1.10E-11 | 1.46E-10 |  | GO:0008324 | cation transmembrane transporter activit... | 297 | 22 | 5.81 | 8.50E-08 | 1.13E-06 |
|  | GO:0004869 | cysteine-type endopeptidase inhibitor ac... | 21 | 10 | 0.5 | 1.40E-11 | 1.68E-10 |  | GO:0020037 | heme binding | 252 | 19 | 4.93 | 5.10E-07 | 5.67E-06 |
|  | GO:0004601 | peroxidase activity | 102 | 18 | 2.44 | 2.80E-11 | 3.05E-10 |  | GO:0016491 | oxidoreductase activity | 1207 | 49 | 23.63 | 5.20E-07 | 5.67E-06 |
|  | GO:0016684 | oxidoreductase activity, acting on perox... | 104 | 18 | 2.48 | 3.90E-11 | 3.90E-10 |  | GO:0046906 | tetrapyrrole binding | 271 | 19 | 5.3 | 1.50E-06 | 1.50E-05 |
|  | GO:0016209 | antioxidant activity | 147 | 20 | 3.51 | 2.90E-10 | 2.67E-09 |  | GO:0016709 | oxidoreductase activity, acting on paire... | 95 | 11 | 1.86 | 2.40E-06 | 2.21E-05 |
|  | GO:0045735 | nutrient reservoir activity | 31 | 10 | 0.74 | 1.50E-09 | 1.28E-08 |  | GO:0015179 | L-amino acid transmembrane transporter a... | 22 | 6 | 0.43 | 3.00E-06 | 2.57E-05 |
|  | GO:0004867 | serine-type endopeptidase inhibitor acti... | 18 | 7 | 0.43 | 1.00E-07 | 7.99E-07 |  | GO:0015291 | secondary active transmembrane transport... | 238 | 17 | 4.66 | 4.30E-06 | 3.44E-05 |
|  | GO:0030145 | manganese ion binding | 32 | 8 | 0.76 | 6.10E-07 | 4.57E-06 |  | GO:0005342 | organic acid transmembrane transporter a... | 111 | 11 | 2.17 | 1.10E-05 | 7.75E-05 |
|  | GO:0030234 | enzyme regulator activity | 259 | 21 | 6.19 | 1.00E-06 | 7.05E-06 |  | GO:0046943 | carboxylic acid transmembrane transporte... | 111 | 11 | 2.17 | 1.10E-05 | 7.75E-05 |
|  | GO:0098772 | molecular function regulator | 320 | 23 | 7.64 | 2.50E-06 | 1.66E-05 |  | GO:0022804 | active transmembrane transporter activit... | 400 | 22 | 7.83 | 1.20E-05 | 7.99E-05 |
|  | GO:0016614 | oxidoreductase activity, acting on CH-OH... | 238 | 18 | 5.68 | 1.60E-05 | 0.000101 |  | GO:0015175 | neutral amino acid transmembrane transpo... | 18 | 5 | 0.35 | 1.90E-05 | 0.00012 |
|  | GO:0004568 | chitinase activity | 26 | 6 | 0.62 | 2.70E-05 | 0.000162 |  | GO:0046527 | glucosyltransferase activity | 142 | 12 | 2.78 | 2.20E-05 | 0.000132 |
|  | GO:0043169 | cation binding | 2926 | 97 | 69.89 | 0.00017 | 0.00097 |  | GO:0046873 | metal ion transmembrane transporter acti... | 151 | 12 | 2.96 | 4.10E-05 | 0.000234 |
|  | GO:0046872 | metal ion binding | 2905 | 96 | 69.39 | 0.00021 | 0.001144 |  | GO:0015171 | amino acid transmembrane transporter act... | 68 | 8 | 1.33 | 5.10E-05 | 0.000276 |
|  | GO:0008271 | secondary active sulfate transmembrane t... | 14 | 4 | 0.33 | 0.00026 | 0.001348 |  | GO:0015077 | monovalent inorganic cation transmembran... | 155 | 12 | 3.03 | 5.30E-05 | 0.000276 |
|  | GO:0004553 | hydrolase activity, hydrolyzing O-glycos... | 270 | 17 | 6.45 | 0.00027 | 0.001348 |  | GO:0035251 | UDP-glucosyltransferase activity | 111 | 10 | 2.17 | 6.20E-05 | 0.000302 |
|  | GO:0015116 | sulfate transmembrane transporter activi... | 15 | 4 | 0.36 | 0.00035 | 0.001678 |  | GO:0022890 | inorganic cation transmembrane transport... | 264 | 16 | 5.17 | 6.30E-05 | 0.000302 |
|  | GO:0004604 | phosphoadenylyl-sulfate reductase (thior... | 2 | 2 | 0.05 | 0.00057 | 0.00244 |  | GO:0008514 | organic anion transmembrane transporter ... | 139 | 11 | 2.72 | 8.90E-05 | 0.00041 |
|  | GO:0009973 | adenylyl-sulfate reductase activity | 2 | 2 | 0.05 | 0.00057 | 0.00244 |  | GO:0080044 | quercetin 7-O-glucosyltransferase activi... | 26 | 5 | 0.51 | 0.00013 | 0.000577 |
|  | GO:0033741 | adenylyl-sulfate reductase (glutathione)... | 2 | 2 | 0.05 | 0.00057 | 0.00244 |  | GO:0052638 | indole-3-butyrate beta-glucosyltransfera... | 6 | 3 | 0.12 | 0.00014 | 0.000599 |
|  | GO:0050662 | coenzyme binding | 456 | 23 | 10.89 | 0.0006 | 0.00248 |  | GO:0080043 | quercetin 3-O-glucosyltransferase activi... | 27 | 5 | 0.53 | 0.00016 | 0.000661 |
|  | GO:0030246 | carbohydrate binding | 274 | 16 | 6.54 | 0.00091 | 0.003635 |  | GO:0008509 | anion transmembrane transporter activity | 208 | 13 | 4.07 | 0.00023 | 0.000919 |
| **CC** | GO:0005618 | cell wall | 452 | 36 | 9.08 | 1.10E-12 | 6.32E-11 |  | GO:0016021 | integral component of membrane | 3023 | 83 | 53.56 | 5.50E-06 | 0.000659 |
|  | GO:0030312 | external encapsulating structure | 452 | 36 | 9.08 | 1.10E-12 | 6.32E-11 |  | GO:0031224 | intrinsic component of membrane | 3164 | 83 | 56.06 | 3.40E-05 | 0.002037 |
|  | GO:0048046 | apoplast | 226 | 20 | 4.54 | 2.80E-08 | 1.07E-06 |  | GO:0044425 | membrane part | 3477 | 85 | 61.61 | 0.00036 | 0.014382 |
|  | GO:0071944 | cell periphery | 2556 | 82 | 51.34 | 3.00E-06 | 8.62E-05 |  | GO:0005886 | plasma membrane | 2156 | 56 | 38.2 | 0.00145 | 0.043445 |
|  | GO:0009530 | primary cell wall | 3 | 2 | 0.06 | 0.0012 | 0.027573 |  |  |  |  |  |  |  |  |

**Supplementary Materials Table S6.** Gene ontology enrichment of DEGs identified in WL-S in Rec.

| **up-regulated** | | | | | | | |  | **down-regulated** | | | | | | |
| --- | --- | --- | --- | --- | --- | --- | --- | --- | --- | --- | --- | --- | --- | --- | --- |
| BP | GO.ID | Term | Annotated | Significant | Expected | classicFisher | qvalue |  | GO.ID | Term | Annotated | Significant | Expected | classicFisher | qvalue |
|  | GO:0042737 | drug catabolic process | 196 | 47 | 11.65 | 6.40E-17 | 7.67E-15 |  | GO:1901700 | response to oxygen-containing compound | 1225 | 160 | 75.62 | 2.60E-21 | 3.12E-19 |
|  | GO:0055114 | oxidation-reduction process | 990 | 123 | 58.82 | 6.80E-16 | 4.07E-14 |  | GO:0042221 | response to chemical | 2137 | 230 | 131.92 | 9.20E-20 | 4.39E-18 |
|  | GO:0042743 | hydrogen peroxide metabolic process | 101 | 32 | 6 | 1.20E-15 | 4.79E-14 |  | GO:0010033 | response to organic substance | 1595 | 187 | 98.46 | 1.10E-19 | 4.39E-18 |
|  | GO:0042744 | hydrogen peroxide catabolic process | 77 | 27 | 4.57 | 1.30E-14 | 3.90E-13 |  | GO:0010200 | response to chitin | 114 | 38 | 7.04 | 1.30E-18 | 3.90E-17 |
|  | GO:0017001 | antibiotic catabolic process | 87 | 28 | 5.17 | 4.90E-14 | 1.17E-12 |  | GO:0042493 | response to drug | 426 | 74 | 26.3 | 2.00E-16 | 4.79E-15 |
|  | GO:0072593 | reactive oxygen species metabolic proces... | 154 | 33 | 9.15 | 7.80E-11 | 1.40E-09 |  | GO:0050896 | response to stimulus | 4341 | 373 | 267.99 | 3.20E-16 | 6.39E-15 |
|  | GO:0051187 | cofactor catabolic process | 107 | 27 | 6.36 | 8.20E-11 | 1.40E-09 |  | GO:0009719 | response to endogenous stimulus | 1354 | 157 | 83.59 | 6.90E-16 | 1.18E-14 |
|  | GO:0016999 | antibiotic metabolic process | 175 | 34 | 10.4 | 6.50E-10 | 9.74E-09 |  | GO:0009725 | response to hormone | 1319 | 153 | 81.43 | 1.70E-15 | 2.55E-14 |
|  | GO:0000272 | polysaccharide catabolic process | 129 | 28 | 7.66 | 1.60E-09 | 2.13E-08 |  | GO:0009644 | response to high light intensity | 52 | 22 | 3.21 | 8.40E-14 | 1.12E-12 |
|  | GO:0050896 | response to stimulus | 4341 | 329 | 257.91 | 1.20E-08 | 1.44E-07 |  | GO:0010243 | response to organonitrogen compound | 207 | 43 | 12.78 | 1.10E-12 | 1.32E-11 |
|  | GO:0071554 | cell wall organization or biogenesis | 487 | 61 | 28.93 | 2.00E-08 | 2.18E-07 |  | GO:0006952 | defense response | 1065 | 122 | 65.75 | 5.70E-12 | 6.21E-11 |
|  | GO:0051704 | multi-organism process | 1074 | 107 | 63.81 | 4.10E-08 | 4.09E-07 |  | GO:0009723 | response to ethylene | 257 | 47 | 15.87 | 1.20E-11 | 1.20E-10 |
|  | GO:0009607 | response to biotic stimulus | 791 | 83 | 47 | 1.90E-07 | 1.75E-06 |  | GO:0006721 | terpenoid metabolic process | 132 | 32 | 8.15 | 1.30E-11 | 1.20E-10 |
|  | GO:0071555 | cell wall organization | 356 | 46 | 21.15 | 4.80E-07 | 4.11E-06 |  | GO:1901698 | response to nitrogen compound | 259 | 47 | 15.99 | 1.60E-11 | 1.36E-10 |
|  | GO:0045229 | external encapsulating structure organiz... | 374 | 47 | 22.22 | 8.10E-07 | 6.47E-06 |  | GO:0001101 | response to acid chemical | 937 | 110 | 57.84 | 1.70E-11 | 1.36E-10 |
|  | GO:0016052 | carbohydrate catabolic process | 212 | 32 | 12.6 | 9.70E-07 | 7.27E-06 |  | GO:0016114 | terpenoid biosynthetic process | 119 | 30 | 7.35 | 2.00E-11 | 1.50E-10 |
|  | GO:0017144 | drug metabolic process | 594 | 65 | 35.29 | 1.10E-06 | 7.75E-06 |  | GO:0009607 | response to biotic stimulus | 791 | 97 | 48.83 | 2.70E-11 | 1.90E-10 |
|  | GO:0006979 | response to oxidative stress | 303 | 40 | 18 | 1.60E-06 | 1.07E-05 |  | GO:0006950 | response to stress | 2583 | 234 | 159.46 | 3.00E-11 | 2.00E-10 |
|  | GO:0051707 | response to other organism | 723 | 74 | 42.96 | 2.40E-06 | 1.51E-05 |  | GO:0009642 | response to light intensity | 93 | 25 | 5.74 | 2.10E-10 | 1.32E-09 |
|  | GO:0042221 | response to chemical | 2137 | 174 | 126.97 | 3.10E-06 | 1.86E-05 |  | GO:0043207 | response to external biotic stimulus | 730 | 88 | 45.07 | 6.10E-10 | 3.60E-09 |
|  | GO:0043207 | response to external biotic stimulus | 730 | 74 | 43.37 | 3.40E-06 | 1.94E-05 |  | GO:0071495 | cellular response to endogenous stimulus | 885 | 101 | 54.63 | 6.30E-10 | 3.60E-09 |
|  | GO:0005975 | carbohydrate metabolic process | 767 | 75 | 45.57 | 1.10E-05 | 5.99E-05 |  | GO:0046677 | response to antibiotic | 222 | 40 | 13.7 | 6.80E-10 | 3.70E-09 |
|  | GO:0009719 | response to endogenous stimulus | 1354 | 117 | 80.45 | 1.40E-05 | 6.91E-05 |  | GO:0032870 | cellular response to hormone stimulus | 848 | 97 | 52.35 | 1.30E-09 | 6.49E-09 |
|  | GO:0001906 | cell killing | 11 | 6 | 0.65 | 1.50E-05 | 6.91E-05 |  | GO:0009755 | hormone-mediated signaling pathway | 777 | 91 | 47.97 | 1.30E-09 | 6.49E-09 |
|  | GO:0031640 | killing of cells of other organism | 11 | 6 | 0.65 | 1.50E-05 | 6.91E-05 |  | GO:0042542 | response to hydrogen peroxide | 54 | 18 | 3.33 | 1.60E-09 | 7.67E-09 |
|  | GO:0044364 | disruption of cells of other organism | 11 | 6 | 0.65 | 1.50E-05 | 6.91E-05 |  | GO:0071310 | cellular response to organic substance | 943 | 103 | 58.21 | 4.80E-09 | 2.21E-08 |
|  | GO:0006952 | defense response | 1065 | 96 | 63.28 | 1.80E-05 | 7.99E-05 |  | GO:0051707 | response to other organism | 723 | 84 | 44.63 | 9.10E-09 | 4.04E-08 |
|  | GO:0009605 | response to external stimulus | 1026 | 92 | 60.96 | 3.40E-05 | 0.000146 |  | GO:0006720 | isoprenoid metabolic process | 169 | 32 | 10.43 | 1.00E-08 | 4.28E-08 |
|  | GO:0009725 | response to hormone | 1319 | 112 | 78.37 | 4.80E-05 | 0.000198 |  | GO:0023052 | signaling | 1474 | 143 | 91 | 1.10E-08 | 4.39E-08 |
|  | GO:0080092 | regulation of pollen tube growth | 13 | 6 | 0.77 | 5.20E-05 | 0.000208 |  | GO:0007165 | signal transduction | 1448 | 141 | 89.39 | 1.10E-08 | 4.39E-08 |
| **MF** | GO:0020037 | heme binding | 252 | 55 | 15.45 | 5.20E-17 | 6.23E-15 |  | GO:0140110 | transcription regulator activity | 1127 | 147 | 67.3 | 7.10E-21 | 8.39E-19 |
|  | GO:0046906 | tetrapyrrole binding | 271 | 55 | 16.61 | 1.50E-15 | 8.99E-14 |  | GO:0003700 | DNA-binding transcription factor activit... | 1065 | 141 | 63.6 | 1.40E-20 | 8.39E-19 |
|  | GO:0004601 | peroxidase activity | 102 | 32 | 6.25 | 4.10E-15 | 1.64E-13 |  | GO:0043565 | sequence-specific DNA binding | 632 | 80 | 37.74 | 7.80E-11 | 3.12E-09 |
|  | GO:0016684 | oxidoreductase activity, acting on perox... | 104 | 32 | 6.37 | 7.70E-15 | 2.31E-13 |  | GO:0003677 | DNA binding | 1797 | 162 | 107.31 | 1.50E-08 | 4.49E-07 |
|  | GO:0016209 | antioxidant activity | 147 | 38 | 9.01 | 1.30E-14 | 3.12E-13 |  | GO:0046527 | glucosyltransferase activity | 142 | 25 | 8.48 | 9.40E-07 | 2.25E-05 |
|  | GO:0004866 | endopeptidase inhibitor activity | 41 | 20 | 2.51 | 3.50E-14 | 5.99E-13 |  | GO:0016762 | xyloglucan:xyloglucosyl transferase acti... | 27 | 9 | 1.61 | 1.60E-05 | 3.20E-04 |
|  | GO:0030414 | peptidase inhibitor activity | 41 | 20 | 2.51 | 3.50E-14 | 5.99E-13 |  | GO:0016758 | transferase activity, transferring hexos... | 300 | 36 | 17.91 | 4.80E-05 | 8.22E-04 |
|  | GO:0061135 | endopeptidase regulator activity | 42 | 20 | 2.57 | 6.20E-14 | 9.29E-13 |  | GO:0016705 | oxidoreductase activity, acting on paire... | 235 | 29 | 14.03 | 1.60E-04 | 2.29E-03 |
|  | GO:0016491 | oxidoreductase activity | 1207 | 138 | 73.98 | 1.10E-13 | 1.32E-12 |  | GO:0016757 | transferase activity, transferring glyco... | 420 | 44 | 25.08 | 1.90E-04 | 2.29E-03 |
|  | GO:0061134 | peptidase regulator activity | 43 | 20 | 2.64 | 1.10E-13 | 1.32E-12 |  | GO:0018488 | aryl-aldehyde oxidase activity | 3 | 3 | 0.18 | 2.10E-04 | 2.29E-03 |
|  | GO:0048037 | cofactor binding | 858 | 104 | 52.59 | 6.10E-12 | 6.65E-11 |  | GO:0050302 | indole-3-acetaldehyde oxidase activity | 3 | 3 | 0.18 | 2.10E-04 | 2.29E-03 |
|  | GO:0004857 | enzyme inhibitor activity | 121 | 26 | 7.42 | 1.40E-08 | 1.40E-07 |  | GO:0004311 | farnesyltranstransferase activity | 7 | 4 | 0.42 | 3.80E-04 | 3.25E-03 |
|  | GO:0004553 | hydrolase activity, hydrolyzing O-glycos... | 270 | 41 | 16.55 | 5.80E-08 | 5.35E-07 |  | GO:0004337 | geranyltranstransferase activity | 7 | 4 | 0.42 | 3.80E-04 | 3.25E-03 |
|  | GO:0004869 | cysteine-type endopeptidase inhibitor ac... | 21 | 10 | 1.29 | 1.30E-07 | 1.11E-06 |  | GO:0051087 | chaperone binding | 39 | 9 | 2.33 | 3.80E-04 | 3.25E-03 |
|  | GO:0016798 | hydrolase activity, acting on glycosyl b... | 304 | 43 | 18.63 | 2.20E-07 | 1.76E-06 |  | GO:0005506 | iron ion binding | 197 | 24 | 11.76 | 6.80E-04 | 5.43E-03 |
|  | GO:0004867 | serine-type endopeptidase inhibitor acti... | 18 | 9 | 1.1 | 3.40E-07 | 2.55E-06 |  | GO:0004031 | aldehyde oxidase activity | 4 | 3 | 0.24 | 8.10E-04 | 5.71E-03 |
|  | GO:0004497 | monooxygenase activity | 186 | 28 | 11.4 | 8.70E-06 | 6.13E-05 |  | GO:0016623 | oxidoreductase activity, acting on the a... | 4 | 3 | 0.24 | 8.10E-04 | 0.00571 |
|  | GO:0004650 | polygalacturonase activity | 20 | 8 | 1.23 | 1.20E-05 | 7.99E-05 |  | GO:0035251 | UDP-glucosyltransferase activity | 111 | 16 | 6.63 | 8.80E-04 | 0.005859 |
|  | GO:0016705 | oxidoreductase activity, acting on paire... | 235 | 32 | 14.4 | 1.70E-05 | 0.000107 |  | GO:0005516 | calmodulin binding | 195 | 23 | 11.64 | 1.36E-03 | 0.008579 |
|  | GO:0016829 | lyase activity | 294 | 37 | 18.02 | 2.30E-05 | 0.000138 |  | GO:0016709 | oxidoreductase activity, acting on paire... | 95 | 14 | 5.67 | 1.46E-03 | 0.008749 |
|  | GO:0030246 | carbohydrate binding | 274 | 34 | 16.79 | 6.60E-05 | 0.000376 |  | GO:0004497 | monooxygenase activity | 186 | 22 | 11.11 | 1.65E-03 | 0.009417 |
|  | GO:0045735 | nutrient reservoir activity | 31 | 9 | 1.9 | 6.90E-05 | 0.000376 |  | GO:0008194 | UDP-glycosyltransferase activity | 199 | 23 | 11.88 | 1.78E-03 | 0.009697 |
|  | GO:0008271 | secondary active sulfate transmembrane t... | 14 | 6 | 0.86 | 0.0001 | 0.000521 |  | GO:0033772 | flavonoid 3',5'-hydroxylase activity | 2 | 2 | 0.12 | 3.56E-03 | 0.017738 |
|  | GO:0098772 | molecular function regulator | 320 | 37 | 19.61 | 0.00014 | 0.000699 |  | GO:0050474 | (S)-norcoclaurine synthase activity | 2 | 2 | 0.12 | 3.56E-03 | 0.017738 |
|  | GO:0005544 | calcium-dependent phospholipid binding | 15 | 6 | 0.92 | 0.00016 | 0.000728 |  | GO:0050378 | UDP-glucuronate 4-epimerase activity | 6 | 3 | 0.36 | 0.0037 | 0.017738 |
|  | GO:0015116 | sulfate transmembrane transporter activi... | 15 | 6 | 0.92 | 0.00016 | 0.000728 |  | GO:0004722 | protein serine/threonine phosphatase act... | 75 | 11 | 4.48 | 0.00472 | 0.021757 |
|  | GO:0046593 | mandelonitrile lyase activity | 10 | 5 | 0.61 | 0.00017 | 0.000728 |  | GO:0038023 | signaling receptor activity | 217 | 23 | 12.96 | 0.00533 | 0.023659 |
|  | GO:0005509 | calcium ion binding | 197 | 26 | 12.07 | 0.00017 | 0.000728 |  | GO:0004430 | 1-phosphatidylinositol 4-kinase activity | 7 | 3 | 0.42 | 0.00619 | 0.025582 |
|  | GO:0003824 | catalytic activity | 6947 | 472 | 425.8 | 0.0002 | 0.000827 |  | GO:0102229 | amylopectin maltohydrolase activity | 7 | 3 | 0.42 | 0.00619 | 0.025582 |
|  | GO:0102483 | scopolin beta-glucosidase activity | 22 | 7 | 1.35 | 0.00024 | 0.000959 |  | GO:0070696 | transmembrane receptor protein serine/th... | 14 | 4 | 0.84 | 7.80E-03 | 0.031161 |
| **CC** | GO:0071944 | cell periphery | 2556 | 245 | 148.18 | 2.60E-18 | 2.99E-16 |  | ----- |  |  |  |  |  |  |
|  | GO:0005618 | cell wall | 452 | 75 | 26.2 | 5.30E-17 | 2.03E-15 |  |  |  |  |  |  |  |  |
|  | GO:0030312 | external encapsulating structure | 452 | 75 | 26.2 | 5.30E-17 | 2.03E-15 |  |  |  |  |  |  |  |  |
|  | GO:0048046 | apoplast | 226 | 33 | 13.1 | 8.20E-07 | 2.36E-05 |  |  |  |  |  |  |  |  |
|  | GO:0005886 | plasma membrane | 2156 | 167 | 124.99 | 2.30E-05 | 0.000528 |  |  |  |  |  |  |  |  |
|  | GO:0042555 | MCM complex | 8 | 5 | 0.46 | 3.10E-05 | 0.000594 |  |  |  |  |  |  |  |  |
|  | GO:0009530 | primary cell wall | 3 | 3 | 0.17 | 0.00019 | 0.003118 |  |  |  |  |  |  |  |  |
|  | GO:0090406 | pollen tube | 31 | 7 | 1.8 | 0.00165 | 0.023696 |  |  |  |  |  |  |  |  |
|  | GO:0016021 | integral component of membrane | 3023 | 207 | 175.25 | 0.00289 | 0.035439 |  |  |  |  |  |  |  |  |
|  | GO:0031224 | intrinsic component of membrane | 3164 | 215 | 183.43 | 0.00339 | 0.035439 |  |  |  |  |  |  |  |  |
|  | GO:0009505 | plant-type cell wall | 166 | 19 | 9.62 | 0.00347 | 0.035439 |  |  |  |  |  |  |  |  |
|  | GO:0000347 | THO complex | 12 | 4 | 0.7 | 0.00381 | 0.035439 |  |  |  |  |  |  |  |  |
|  | GO:0005887 | integral component of plasma membrane | 132 | 16 | 7.65 | 0.00401 | 0.035439 |  |  |  |  |  |  |  |  |
|  | GO:0044421 | extracellular region part | 173 | 19 | 10.03 | 0.00546 | 0.043581 |  |  |  |  |  |  |  |  |
|  | GO:0032541 | cortical endoplasmic reticulum | 7 | 3 | 0.41 | 0.00569 | 0.043581 |  |  |  |  |  |  |  |  |
